# Intelligent autonomous treatment of bedwetting using non-invasive wearable advanced mechatronics systems and MEMS sensors: Intelligent autonomous bladder monitoring to treat NE

**DOI:** 10.1007/s11517-019-02091-x

**Published:** 2020-02-24

**Authors:** Kaya Kuru, Darren Ansell, Martin Jones, Benjamin Jon Watkinson, Noreen Caswell, Peter Leather, Andrew Lancaster, Paula Sugden, Eleanor Briggs, Carl Davies, Teik Chooi Oh, Kina Bennett, Christian De Goede

**Affiliations:** 1 School of Engineering, University of Central Lancashire, Fylde Rd, Preston, PR1 2HE UK; 2 University of Central Lancashire, Preston, UK; 3 Lancashire Teaching Hospitals NHS Foundation Trust, Preston, UK

**Keywords:** Bedwetting, Ultrasound, Bladder, Nocturnal enuresis, Advanced mechatronics, MEMS, Wearable health monitoring devices

## Abstract

Post-void alarm systems to monitor bedwetting in nocturnal enuresis (NE) have been deemed unsatisfactory. The aim of this study is to develop a safe, comfortable and non-invasive pre-void wearable alarm and associated technologies using advanced mechatronics. Each stage of development includes patient and public involvement and engagement (PPI). The early stages of the development involved children with and without NE (and parents) who were tested at a hospital under the supervision of physicians, radiologists, psychologists, and nurses. The readings of the wearable device were simultaneously compared with B-mode images and measurements, acquired from a conventional ultrasound device, and were found to correlate highly. The results showed that determining imminent voiding need is viable using non-invasive sensors. Following on from “proof of concept,” a bespoke advanced mechatronics device has been developed. The device houses custom electronics, an ultrasound system, intelligent software, a user-friendly smartphone application, bedside alarm box, and a dedicated undergarment, along with a self-adhesive gel pad—designed to keep the MEMS sensors aligned with the abdomen. Testing of the device with phantoms and volunteers has been successful in determining bladder volume and associated voiding need. Five miniaturised, and therefore more ergonomic, versions of the device are being developed, with an enabled connection to the cloud platform for location independent control and monitoring. Thereafter, the enhanced device will be tested with children with NE at their homes for 14 weeks, to gain feedback relating to wearability and data collection involving the cloud platform.

**Electronic supplementary material:**

The online version of this article (10.1007/s11517-019-02091-x) contains supplementary material, which is available to authorized users.

**Graphical Abstract:**
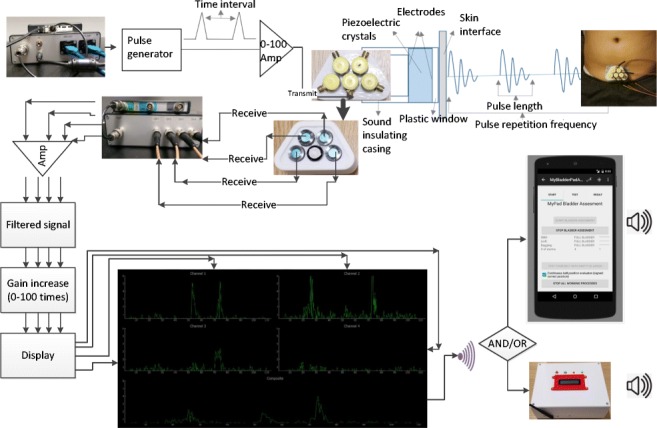
Design of the MyPAD advanced mechatronics system

## Introduction

Nocturnal enuresis (NE), i.e. bedwetting, is the involuntary discharge of urine at night in a child in the absence of congenital or acquired defects of the central nervous system or urinary tract [1]. NE is the most common childhood complaint [2] affecting 15 to 20% of 5-year-old children, 5% of 10-year-old children, and 1 to 2% of people aged 15 years and older [3, 4]. Not only can it effect a child’s life in every aspect negatively, but it is also very stressful for the parents or carers [5, 6]. Additionally, the cost for the family is estimated at £3000 per year. A comprehensive cost analysis along with the negative effects of NE on normal daily routines and social activities can be found in our previous study [1].

Catheterisation remains the gold standard for bladder volume assessment, but it is invasive, uncomfortable to the patient, and introduces the risk of infection and trauma [7]. Therefore, new non-invasive approaches are needed to monitor the bladder and to treat NE. Successful treatment of bedwetting tends to show increased self-esteem for children [8]. A moisture detection alarm as a first-line treatment is recommended for up to 3 months, depending on the dry nights, by both The National Institute for Health and Care Excellence (NICE) [9] and European Society for Paediatric Urology (ESPU) [10]. A number of non-invasive products that can be categorised as (i) bedwetting alarms, (ii) pad and bell alarms, and (iii) bladder scanners currently available in the market to support the monitoring, diagnosis, and treatment of bed-wetting are yet to be found satisfactory in alleviating the predicaments of children with NE [1]. Similarly, medications (e.g. Tricyclics, depression) are associated with potential side effects [11] in addition to their poor performance [12]. This suggests a need to explore alternative modalities. The initial request for innovation in this area, a more optimal device originated from clinicians, parents, and children who were unsatisfied with the performance of the traditional urinary post-void moisture alarms and medications [1] where alarms are considered to be more effective than medications [13].^1^ In this regard, there have been several attempts to find a pre-void solution for NE sufferers [14, 15]. An analysis of these attempts was presented in our previous study [1]. The conclusion drawn is that there is no product in the market that can predict a pre-void occurrence so far.

There is an urgent need to develop an effective and comfortable device that (1) harbours artificial intelligence (AI) techniques to learn and evaluate the dynamic characteristics of the bladder and its surrounding tissues intelligently, (2) has customisable abilities for the children with various types of body morphologies, (3) determines imminent voiding need, and finally (4) provides pre-void alerts accordingly, in order to allow a patient to void in a dignified manner. We analysed the feasibility of such a system in our previous study [1]. That study was developed to explore whether existing technologies could be synchronised, enhanced, and modulated to form an intelligent alarm system that could provide a pre-void warning system for sufferers. More explicitly, in that study, the viability of building, refining, and evaluating a new, safe, comfortable, and non-invasive wearable autonomous intelligent electronic device to monitor the bladder using a single element low-powered low-frequency ultrasound (US) with the help of artificial intelligence (AI) and machine learning (ML) techniques was carried out. The results indicated that customised imminent voiding need, based on the expansion of the bladder, could be determined by applying a single element transducer onto a bladder in an intermittent manner and the acquired results could be improved further with a comfortable non-invasive device by adding several other features. The approaches and techniques determined to cure the NE were patented nationally and internationally [4, 16–20].

The aim in Advanced Mechatronics Systems (AMSs) development is to produce high-quality intelligent autonomous products and maintain a competitive edge through better product performance by forging effective sensing, self-learning, self-optimisation, self-configuration, self-diagnosis, and precise autonomous decision making and actuation. This is performed with no or less human intervention using effective location-independent monitoring, control, and management applications with products [21]. With the advanced wireless communication techniques and improved battery technologies, AMSs are capable of becoming independent and working with other massive AMSs to construct robust, customisable, energy-efficient, autonomous, intelligent, and immersive platforms [21]. Miniaturisation of components and consequently devices using MEMS (Micro-Electro-Mechanical Systems) technology is imperative for ergonomic and functional use. This technology is effectively used in almost every field such as automotive, electronics, medicine, communications, and defense, (e.g. airbag, intelligent tyres, disk drive heads). In this regard, the main objective of this study is to develop a robust, cheaper, more reliable, more flexible, customisable, and effective dry alarm to treat NE and manage bedwetting, which would be more acceptable than any currently available moisture alarms until the child has learned to control the bladder. More explicitly, this study has been carried out to explore whether existing technologies could be synchronised, enhanced, miniaturised, and modulated to form an AMS that could provide a pre-void warning, minimising bedwetting, reaching stable dryness through learning bladder control, and enhancing quality of life for children and families. To clarify the novelty of this paper, the contributions are outlined as follows.

1. To our knowledge, this study is the most comprehensive study in the literature to find an effective solution— i.e. a pre-void alarm system to treat NE involving a cross-disciplinary team, Patient and Public Involvement and Engagement (PPI) in cooperation with various prominent organisations, expert companies, and the AMS technologies.
2. This is the first attempt that explicitly studies a pre-void AMS alarm system to treat NE by forging the features of the edge and cloud platforms, communication technologies, and AI (particularly, reinforcement learning (RL)) and ML to enable the implementation of an automated, multi-functional, autonomous customisable system through self-learning by using transdisciplinary knowledge to address the challenges involved.
3. Bespoke US MEMS sensors specific to this application area have been built and incorporated into the system, and their viability and usability is tested both on phantoms and volunteers, in order to build a more comfortable wearable device.
4. Characteristics of the bladder with respect to liquid consumed and time are analysed in order to create a bespoke application specific to this organ, which can also direct other studies related to the bladder.
5. Bladder volume calculation with respect to the height and width of the bladder using 2D images acquired from a conventional US device has been analysed, which can help other studies related to the volume calculation of the bladder.
6. Morphologies of children are analysed in order to design the comfortable wearable undergarment and the other components, which can direct other studies related to wearable devices designed for children.

The paper is organised as follows. Section 1 outlines the background of the study including a comprehensive state-of-the-art literature on the evolution of NE, and previous and current attempts in order to cure NE. The approaches and techniques performed and the components used in this study are explored in Section 2. The results are presented in Section 3 along with the discussions. Finally, outlining the limitations in Section 4, Section 5 draws conclusions and Section 6 provides directions for potential future works.

## Methods

**Fig. 1:**
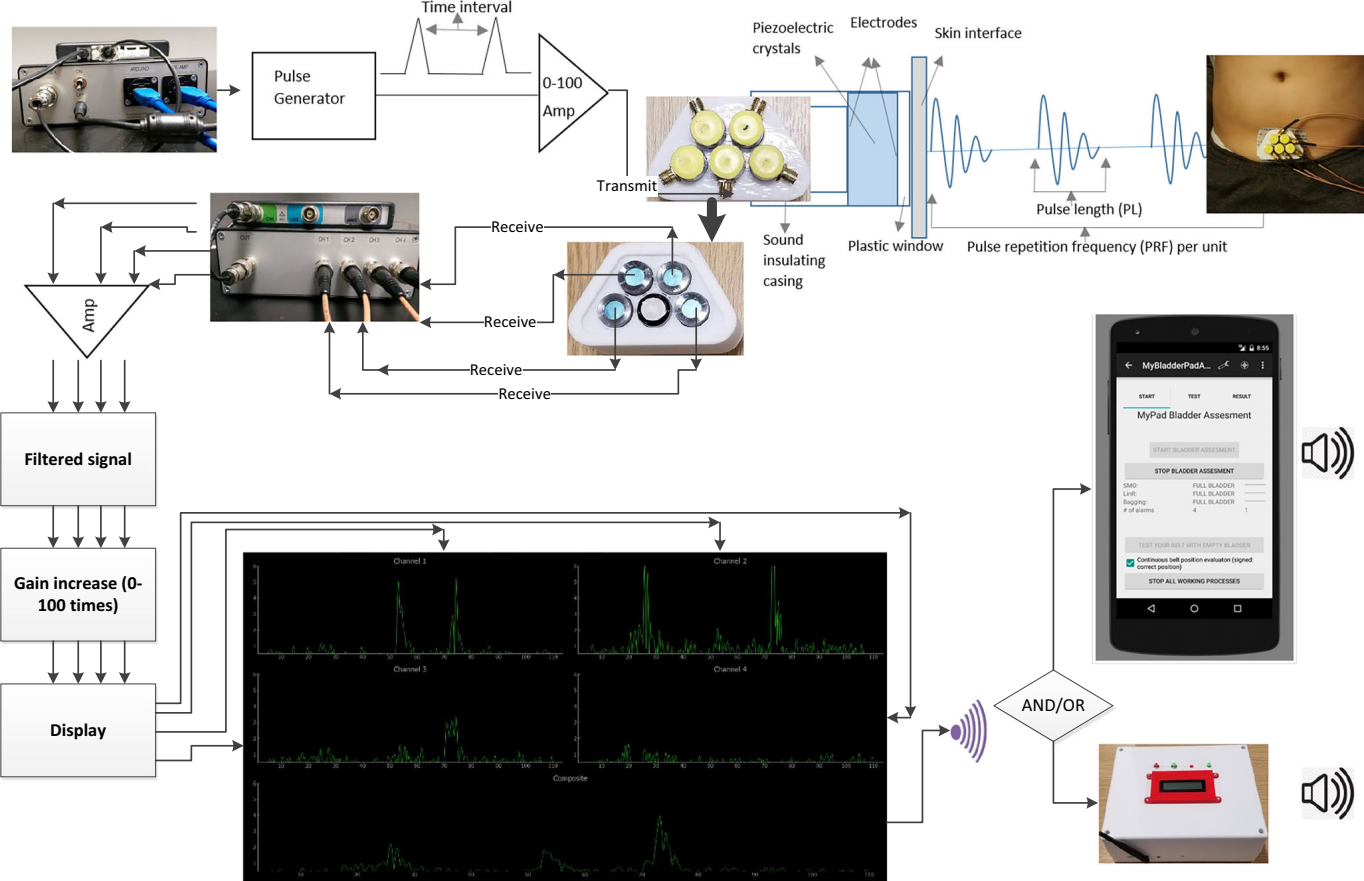
Design of the MyPAD advanced mechatronics system

A non-invasive advanced mechatronics (AM) device which uses multiple US sensors has been developed and tested in this study to cure NE by providing a pre-void alarm. The overall methodology is illustrated in Fig. 1. The sensors are designed to be static as being stuck to the abdomen at the same location supported by an ergonomic undergarment and a sticky gel pad in order to increase the chance of reproducibility of the US echoed pulses with the simplest form of the US technique—A-mode. More explicitly, no US image is acquired where producing a US image requires the scanning of sensors, combination of the scanned slices and reduces the observation of the same image each time based on the changing environmental parameters (e.g. applied angle, scanned surface) resulting in poor reproducibility. The sensors detect “A” mode echoed pulses autonomously to estimate the percentage of filling of bladder compared with the full bladder of urine using various features—e.g. the distance between the anterior and posterior walls of the bladder, amplitudes through the bladder and surrounding tissues, in order to determine the voiding need. The scope and background of the study is explained in Section 2.1 before explaining the techniques, approaches, and technologies utilised in the study in Sections 2.2, 2.3, 2.4, 2.5, 2.6, and 2.7.

### Scope and background of the study

A comprehensive team from various disciplines has been established after the satisfactory results obtained from our feasibility study [1] to build an ergonomic and robust solution for children with NE. Software (SW) and electronics engineers, designers, commercial innovators, health economists, physicians, radiologists, nurses, and psychologists have been brought together in this team. Additionally, the team has established a collaboration with the prominent organisations, ERIC (Education and Resources for improving Childhood Continence) and Lancashire Teaching Hospitals NHS Trust (LTHTR) and the University of Central Lancashire. Patient and public involvement (PPI) is critical to improve the proposed medical device and to ensure the user acceptance of the device. The geometry, wearability, usability, warning method, and optimisation of the device are being iterated through the PPI groups established by LTHTR and our partner charity ERIC. In this regard, children with NE and their families have been included in the study and several workshops have been organised with them in order for us to understand and meet their needs thoroughly in the development of the MyPAD device. Moreover, our collaborator from industry, NovoSound Ltd., that has safely applied their US technology to various industrial fields, has provided us access to a team of experts in ultrasonic technology and related custom electronics.

### Workshops

**Fig. 2:**
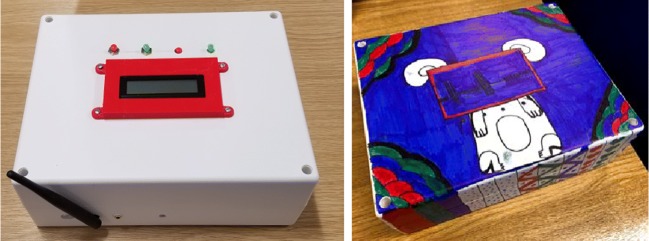
The bed-side alarm box (left) and the box painted by the children (right)

Our MyPAD team has come together with the children and their families several times. One meeting was hosted by ERIC in Bristol, and three children with NE and their families, and one parent whose child did not wish to attend were recruited to attend this focus group/workshop. The main research questions of this workshop were (1) what are the experiences of the families in using enuresis alarms? (2) what do families like about the MyPAD prototype?, and (3) what do families not like about the MyPAD prototype? The bed-side alarm-box painted by the children during the workshop is presented in Fig. 2. The detailed, qualitative results of this workshop are to be published in the near future. The answers to these questions are summarised in relation to the themes as follows. (1) Participants experience of previous alarm use had been mixed, with some experiencing success while others had not. A key theme that seemed to account for the difficulties faced related to sleep, the importance of sleep and the effect of disrupted sleep on daytime functioning, including school work. Families also found that the previous alarms had been loud and unpleasant to wake up to. They had disturbed other family members which was also a challenge. The themes of frustration from parents, coupled with perseverance and hope, contributed to the understanding of the families’ experience of using previous alarms. The families had found it difficult using some alarms and there was a sense of frustration surrounding this. However, they had found products that helped them to manage or deal with the challenges which showed perseverance and resourcefulness. Furthermore, there was a continued effort to address the problem and the families had tried several different alarms, aids, trials, and apps between them to attempt to tackle the problem. Experiences with alarms had also led to positive outcomes particularly for one family whose child had become dry over time with the use of an alarm. (2) The second question of what families liked about MyPAD was best understood through the theme of frustration felt by parents. Many of the sources of frustration such as alarm sounding after voiding and having difficulty understanding the condition parents felt could be helped with MyPAD device. This was due to its ability to ‘see inside the bladder’ to wake the child at the pre-void stage and the data it could provide on bladder capacity. (3) What families did not like about MyPAD mainly related to discussion over the garment and making sure that children felt comfortable and would be happy to wear it. This was mostly encapsulated in the theme of children not wanting to feel different. This will be a challenge for the design team to create something that all children feel comfortable wearing when analysis of this theme showed that children at different ages had different preferences and with the knowledge that child compliance could be an issue for the success of the alarm.

Overall the focus group was incredibly valuable as it enabled the research team to experience the perspective of the families and the challenges and conflicts of managing the condition and using existing alarms. This has improved understanding of the social and environmental challenges that will need to be considered in relation to the MyPAD design and development. The main points to consider are as follows:

- Alarm tones should be as un-intrusive as possible and should be available in both the parents and child’s room, preferably without the use of a phone.
- The device should be comfortable to wear and minimally interrupt the various sleeping positions.
- Device and garment should be acceptable to the child in relation to self- and body-esteem and anxiety issues, which are prevalent in this population.
- Parents might benefit from an information leaflet, provided with the alarm, that includes information on how it works, as a lack of belief in the device can affect motivation.
- MyPAD could be useful for offering an improved understanding of the bladder function.

### Bladder tests on children to reveal the features of the bladder during expansion

**Fig. 3:**
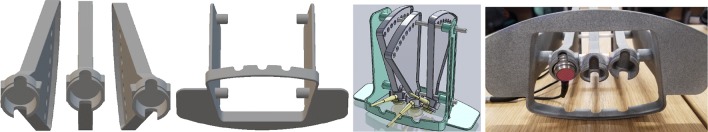
Components of the jig test device

For humans, in supine position, the anterior bladder wall lies at an average depth of about 4 cm, which also depends on the bladder filling stage [22]. In most situations, a full bladder will be closer to the abdominal wall than an empty bladder [22]. The pubic bone between the bladder and the abdomen wall makes it difficult to observe the bladder using a non-invasive approach, as US cannot penetrate through a bone. In children, the bladder lies at a higher level than in adults, above the pubic bone [22], which makes it easier to analyse the bladder compared with adults. When the bladder fills up, it first distends in depth (towards the spine) and then expands in height into the abdominal cavity [22], which makes it easier to analyse the bladder as it fills. The lowest part of the bladder in the standing position, the base, will remain behind the pubic bone. Therefore, orientation of the transducer with an appropriate angle is required to emit US beams into the bladder above the pubic bone in order to detect the greatest distance between the anterior and posterior walls of the bladder, in other words, the largest expansion in the bladder. A 3D print jig test device was developed by our design group to place the probes and adjust the beam angle of the probes as shown in Fig. 3. The beam angle can be adjusted from 5 to 45^∘^ with this jig test device.

**Fig. 4:**
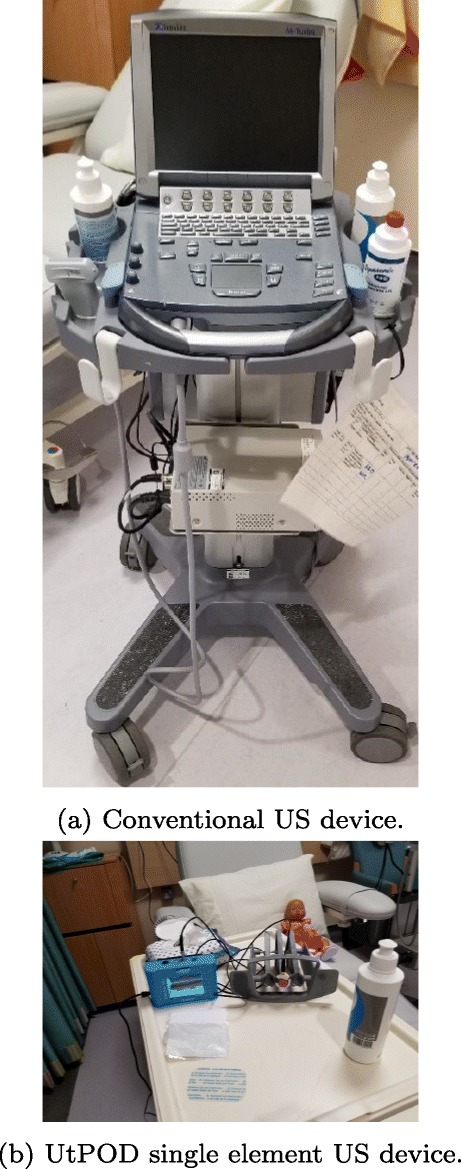
Devices used to test the techniques and approaches

In parallel to the measurements obtained by the new device, a conventional mobile US scan device presented in Fig. 4a was used by a radiologist at the Clinical Research Facility at Royal Preston Hospital to acquire the various levels of urine volume in the bladder by capturing the B-mode images from 6 children between 7 and 14 years old in an intermittent manner (i.e. 20-min intervals) in order to understand the interrelation of the bladder, its filling with urine, and its surrounding media in a dynamic environment. More explicitly, we would like to find out (1) what is the region of interest (ROI) regarding the distance between (i) the sensor (i.e. abdomen) and the anterior wall of the bladder, (ii) the anterior and the posterior walls of the bladder, regarding height and width during the expansion of the bladder, (2) how the thickness of the bladder wall changes with respect to expansion, and (3) what the device listening intervals should be in order to both detect the desired triggering point to wake up the children before bedwetting occurs and save the battery life.

**Fig. 5:**
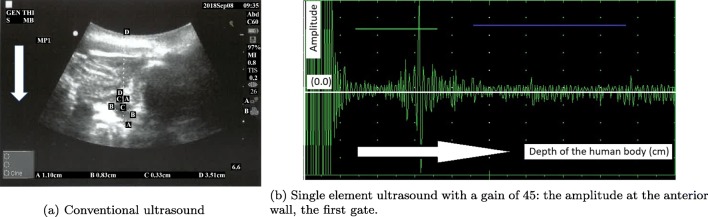
Empty bladder: First trial for the volunteer MP1

**Fig. 6:**
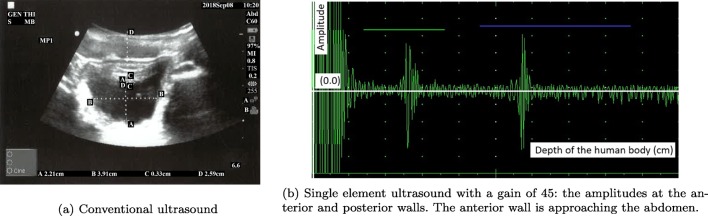
1/2 full bladder: Third trial for the volunteer MP1

**Fig. 7:**
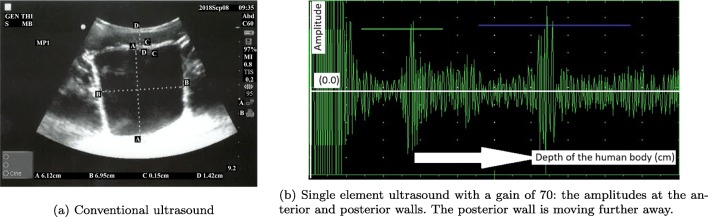
Full bladder: Fifth trial for the volunteer MP1

The children emptied their bladder before starting the test. They consumed liquid (i.e., juice or water) as much as possible during the data collection phase to speed up the procedures and establish the shorter time to fill the bladder. The volume of urine passed was measured using a measuring beaker after the bladder was filled as displayed in Table 1 in the row, ‘Out’. Four values were measured on the B-mode bladder images as displayed in Table 1 in an abstract form, namely height, width, wall thickness of the bladder, and its distance from the probes (i.e. abdomen). The volunteers coded as MP3 and MP4 had drunk a lot of liquid before the trial and most probably they wanted to end the trials rapidly since their full bladder measurements are significantly lower than the normal ranges as explained below in detail and procedures were completed in 45 min ending in voiding. The bladder expands to accommodate the filling volume. All the B-mode images from which the values in Table 1 are formed are in the supplementary materials of this paper. Examples of these images are presented in Figs. 5a, 6a, and 7a as empty, half, and full bladder respectively. Additionally, one echoed pulse was acquired sequentially for each trial using a single element US device and the sensor with the jig test device presented in Fig. 4b. All the A-mode echoed pulses are in the supplementary materials of this paper. Three examples are presented in Figs. 5b, 6b, and 7b. These echoed pulses were captured right after the images in Figs. 5a, 6a, and 7a. The vertical axis corresponds to the amplitude of the echoed pulses whereas the horizontal axis indicates the depth of the human body in which the emitted pulse propagates. The height of the amplitudes decreases, and the thickness of the amplitude increases as the bladder expands and wall thickness reduces. Therefore, the engineer adjusted the gain in order to make the pulses to meet the gates as seen in Fig. 7b even though the echoed pulses were acquired at the desired locations.

**Table 1:**
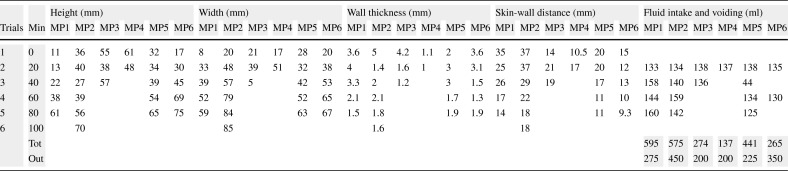
Measured values on the bladder images captured by the conventional US device and voided urine volumes

**Fig. 8:**
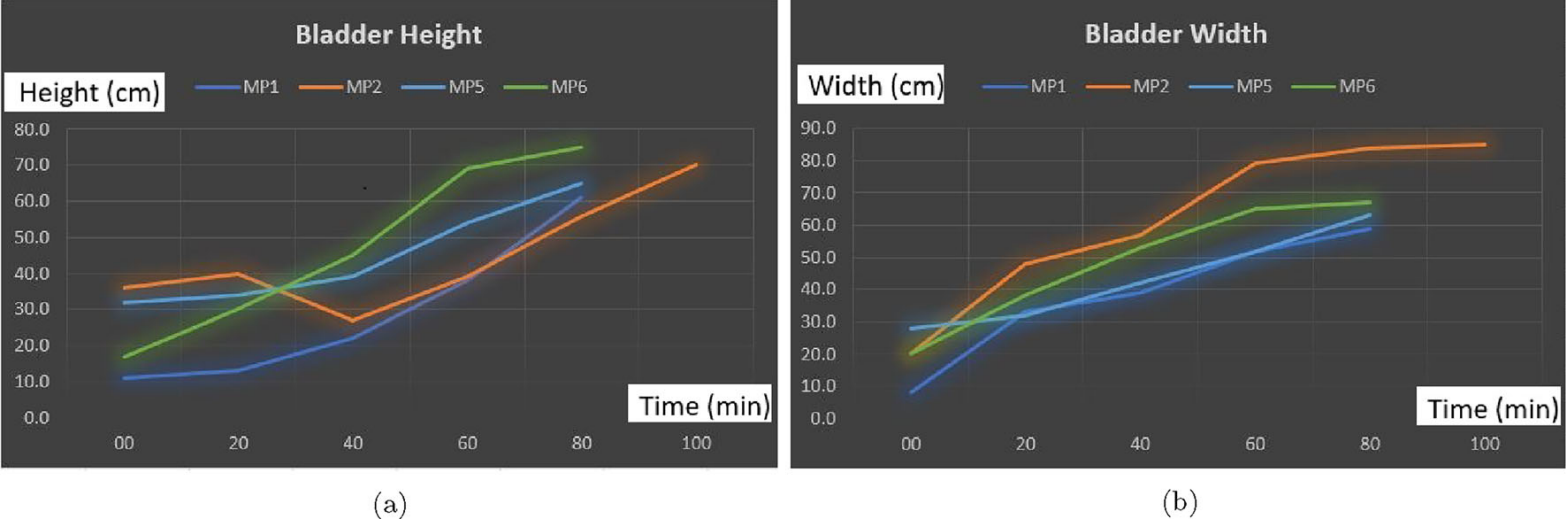
**a** Height and **b** width values of the bladder during the expansion from an empty status to a full status for 6 children in 20-min intervals. The bladder width and height increase as the bladder expands with urine

**Fig. 9:**
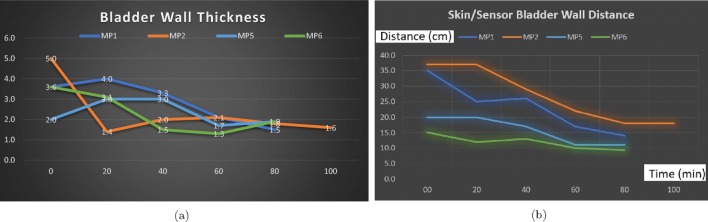
**a** Bladder wall thickness and **b** distance between the sensor and bladder during the expansion from an empty status to a full status for 6 children in 20-min intervals. The thickness of the wall decreases whereas the anterior wall approaches to the abdomen as the bladder expands

The findings of these tests are presented in Figs. 8a, b, and 9 a and b. The ideal degree of the pulse transmission from the probe that gives the greatest distance between the anterior and posterior walls is found to be 25^∘^ by adjusting the beam angle between 5 and 45^∘^ using the jig test device (Fig. 3). Regarding our tests, there is almost a linear expansion for the height and width of the bladder. The height of the bladder may reach to 75 mm and the width may expand up to 85 mm at maximum where the bladder is full. The bladder wall thickness (BWT) starts to decrease above a bladder filling of 50–60 ml ranging from ≈ 5 mm for an empty bladder to ≈ 1 mm for a full bladder. The bladder wall approaches the abdomen from 37.5 mm where it is empty to 10 mm where it is full. With those experiments, we were able to compare the results of our techniques with the results of a conventional US device to reveal the correlation and consequently to determine if our techniques and approaches are working as desired, and most importantly to determine the ROI we need to focus on with respect to the depth of US pulses and echoed pulses generated from the bladder. Additionally, during these tests, we aimed to find out (1) the most appropriate placement of the sensors, and (2) the required measurements in order to tailor the undergarment accordingly, which is explained in the following Section 2.4, while exploring the development of the undergarment regarding the sensors and the gel-pad interface using a 3D print space model.

**Fig. 10:**
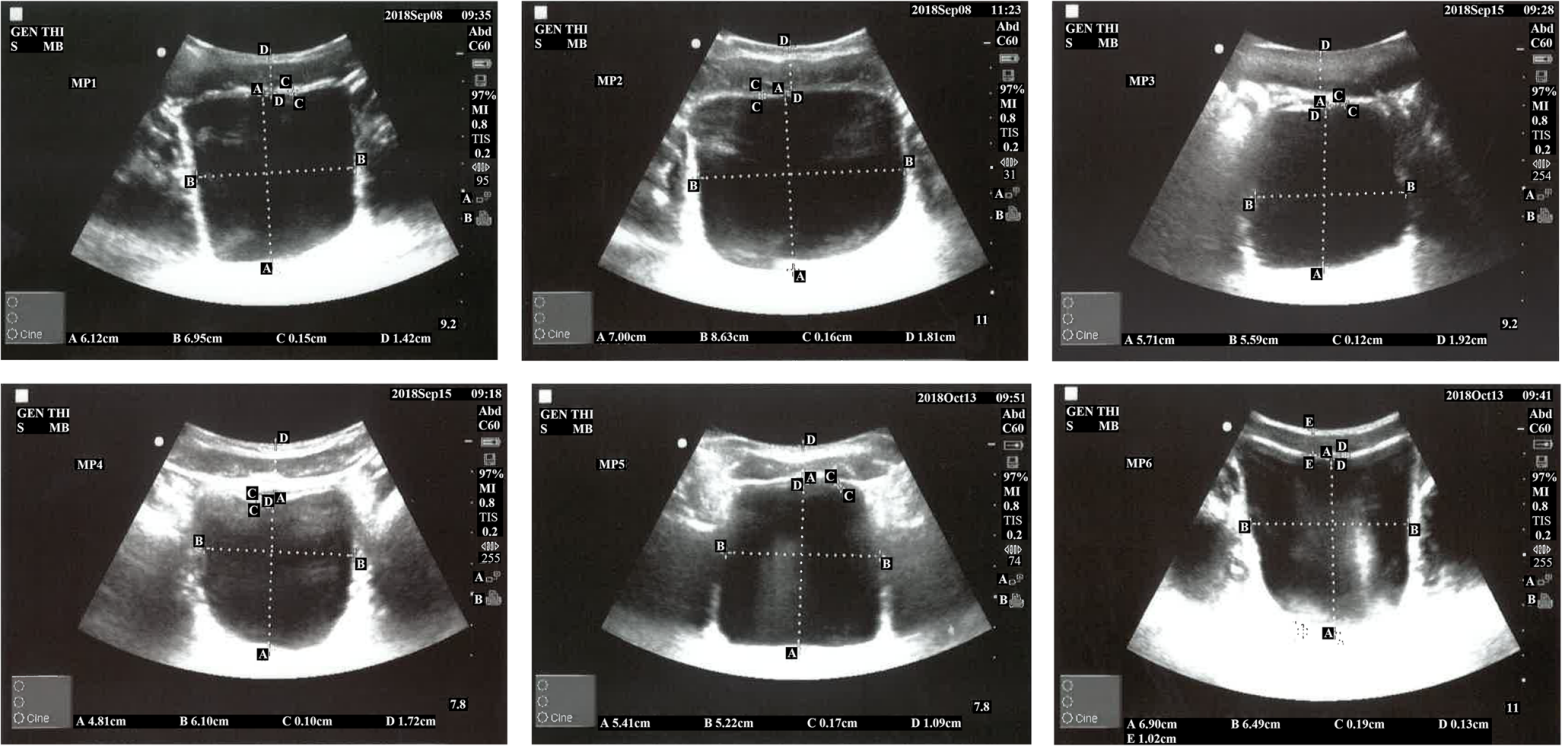
Full bladder images of 6 children from MP1 to MP6

**Fig. 11:**
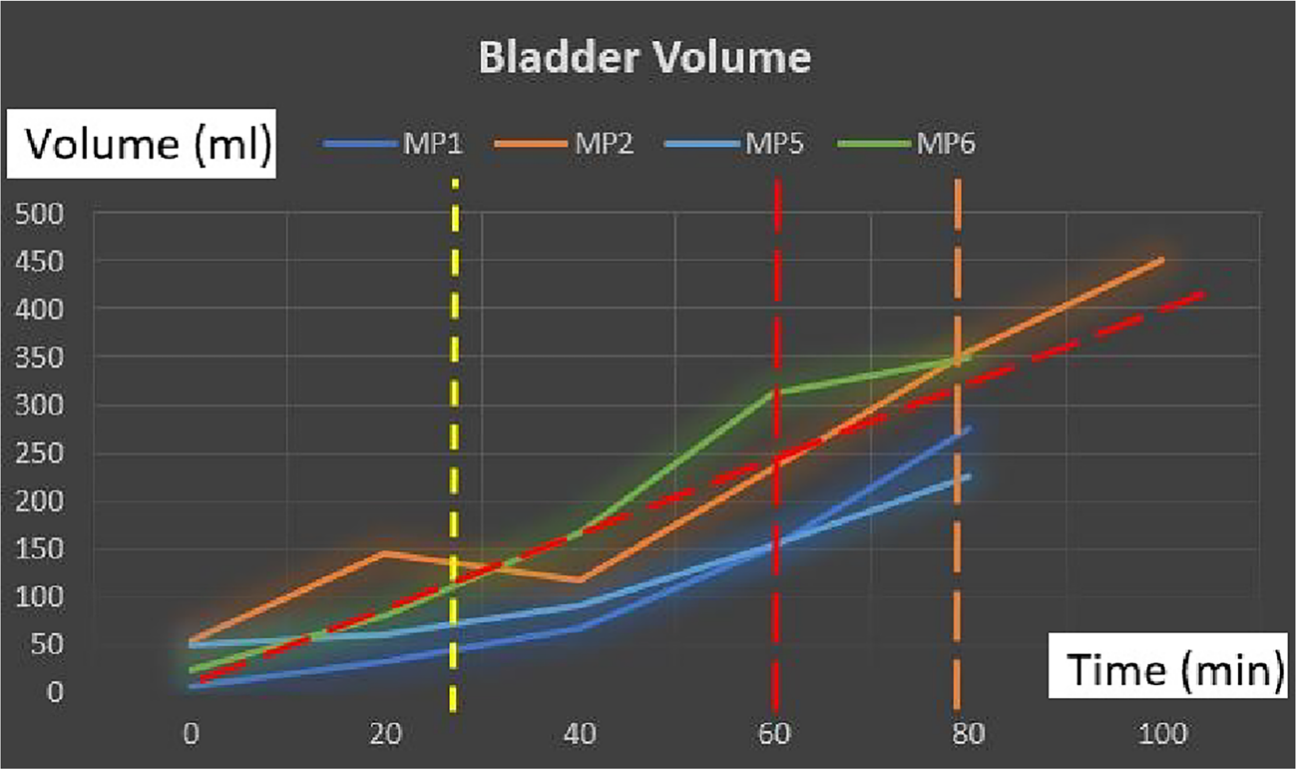
Volume expansion of the bladder: Diagonal red dashed line corresponds to the linear regression of the bladder expansion with respect to time; yellow vertical dashed line indicates the point where the expansion starts, red vertical dashed line shows the point where the voiding need starts for MP1, MP5, and MP6 (7–9-year-old children) whereas the orange vertical dashed line corresponds to the voiding need triggering point for MP2 (14-year-old child)

The full capacity of the bladder volume with respect to the age groups is presented in Table 2 based on the bladder analysis of 3376 children [23]. The bladder volume measurements of the children coded as MP1 and MP6 are within the normal ranges whereas those of MP2 and MP5 are slightly lower than the normal ranges and those of MP3 and MP4 are significantly lower than the normal ranges, either have a small bladder or voided before the bladder was full. We have analysed various volume measurement techniques on US images in the literature [25],[26]. The most commonly used formula is length × height × width × 0.52 on two dimensions where the length is the maximal longitudinal diameter obtained from the sagittal or longitudinal plane, and height and width values are obtained from coronal or frontal plane, in which the ultrasound scanning provides the maximal diameters. In the literature [25],[26], the other formulas for bladder volume calculations also require images from the longitudinal plane and frontal plane at the same time in order to result in better approximate urine volumes. We only have the images from the frontal plane, and therefore, we cannot employ one of these formulas to measure the bladder volume. However, we have the final US measurements and their voided volume measurements as displayed in Table 3 along with the their images in Fig. 10. The customised mapping coefficient for each child is calculated with the formula, Corr. coefficient = (Height × Width)/Voided Vol. The expansion of the bladder with respect to the urine volume for the children is depicted in Fig. 11 using these coefficients in the formula, Urine Volume = (Height × Width)/Corr. coefficient, for each US measurement of the children.

**Table 2:**
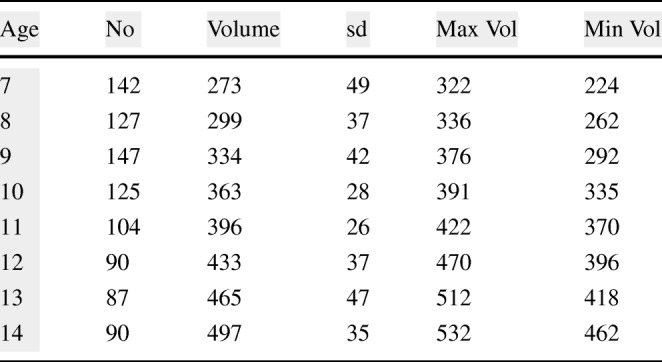
Bladder volume capacity with respect to age [23]

**Table 3:**
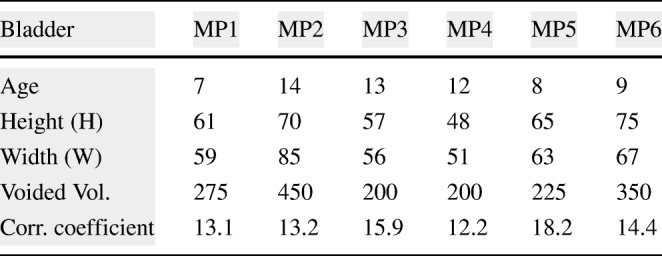
Correlation coefficients of the bladder volume projection for the US measurement of the children

### Development of the ergonomic undergarment

**Fig. 12:**
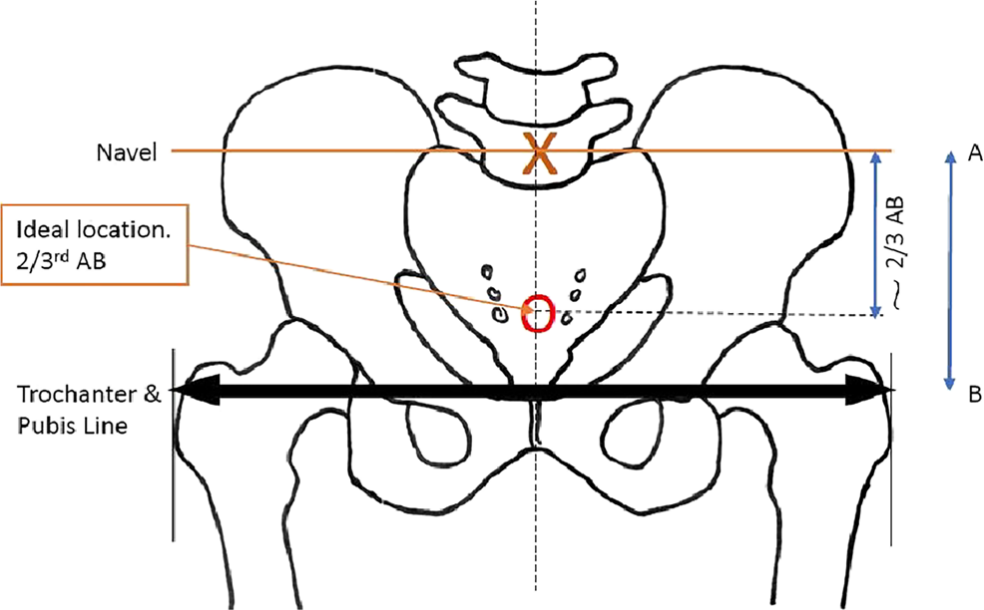
Illustration of the ideal bladder sensing location

A performance critical aspect of this technology is its adoption by the end-user across various psychophysiological requirements. The geometry, wearability, and usability of various undergarment designs were iterated with the PPI groups through various prototype developments. The ideal location of probes has been determined using the measurements taken from 6 children with respect to the horizontal axis between the navel and the pubic bone in the hypo-gastric region as illustrated in Fig. 12.

**Fig. 13:**
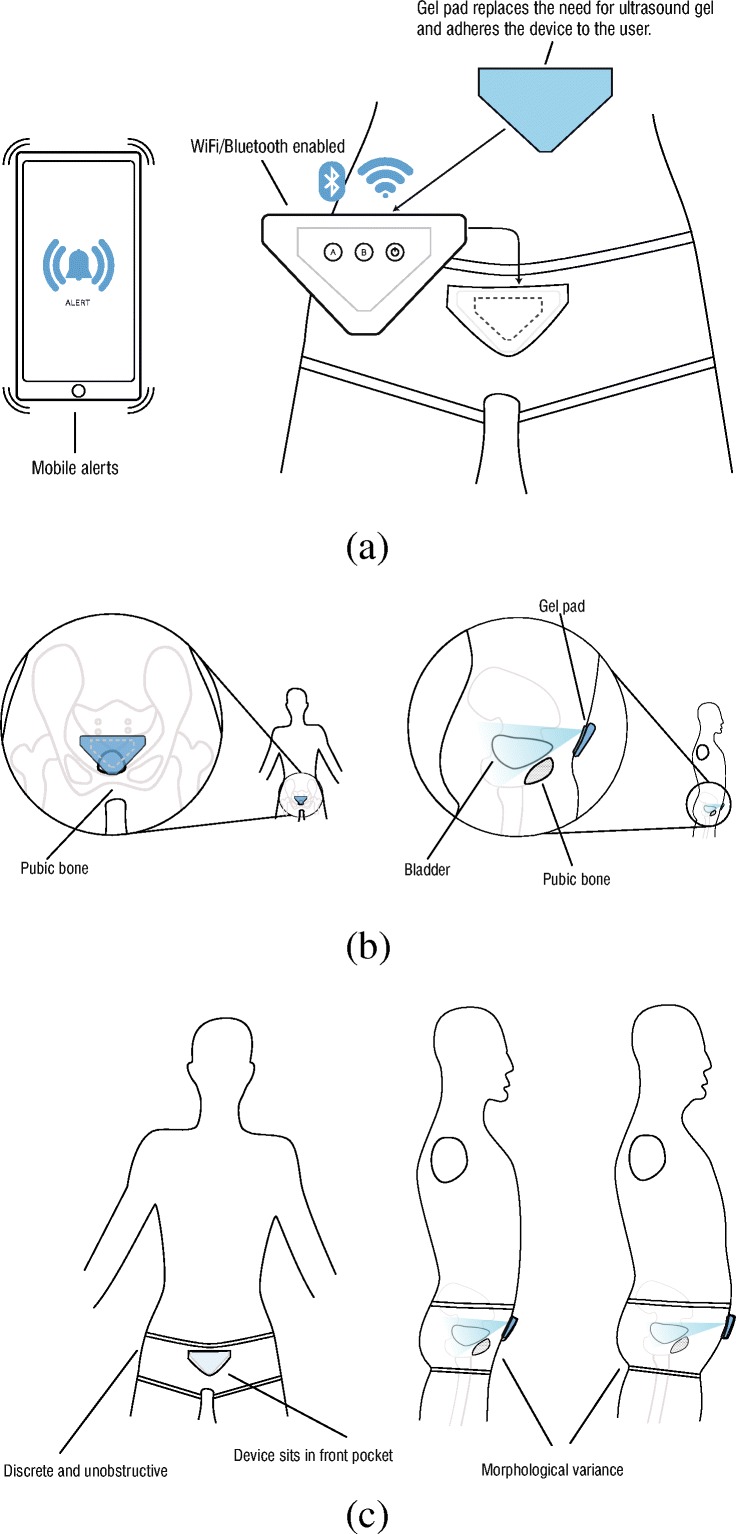
Design concept for a wearable support garment. **a** General framework of the concept. **b** Positioning with respect to bladder. **c** Representation with respect to morphologic types: (i) the devices shape in relation to the pubic region, this will be graded to fit population types. (ii) The garment will provide tension to the rear of the device maintaining contact with the skin. (iii) The inner pocket of the proposed garment has a window which enables the self-adhesive gel pad which is adhered to the body side of the device to protrude through and adhere to the skin

**Fig. 14:**
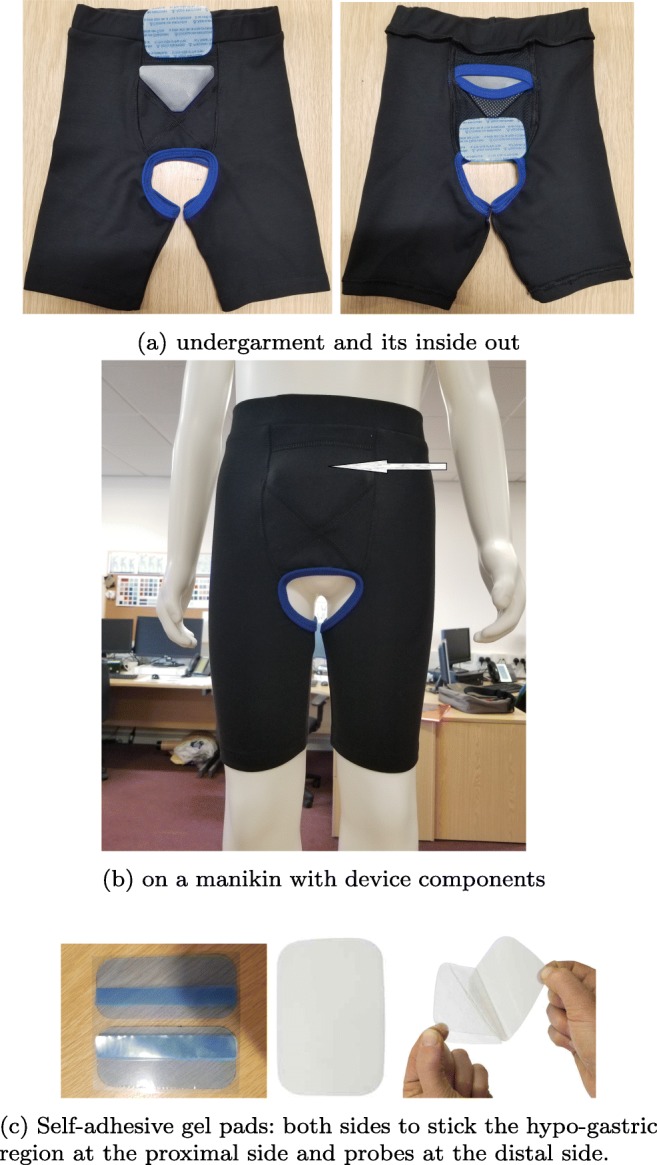
The undergarment along with the space model and the sticky gel pad to be used with the sensors

The most comprehensive study on anthropometry and body morphologies was conducted in 1977 for US Consumer Product Safety Commission based on the analysis of 4127 children [24]. From this report, the waist and upper thigh circumferences with respect to the age groups are presented in Table 4. The measurements taken from the children in our study are presented in Table 5. The waist measurement of the children coded as MP5, MP6, MP8, and MP9 is in the normal ranges whereas the children coded as MP1 and MP7 are not in the normal ranges. Regarding the thigh measurement, the children coded as MP1, MP6, MP8, and MP9 are in the normal ranges whereas the children coded as MP5 and MP7 are not in the normal ranges. Based on the measurements and the feedback from these iterations, our design group at the University of Central Lancashire has worked on a design concept for a wearable support garment along with a gel-containing comfortable absorbent pad for the interface between the transducers and skin to keep the US interfacing component of the device in firm contact with the body, and in an optimum position for obtaining desired bladder measurements as illustrated in Fig. 13. The undergarment developed is presented in Fig. 14. It has been tailored for children to be able to void without removing the undergarment and the components of the MyPAD device from their bodies. Children can wear their own underwear over the MyPAD garment. In addition, it can be separated from these components to be washed separately. More specifically, this approach provides two critical advantageous: (i) ease of application/use by a non-technical person to the body, by user, or carer offering instant accurate location of the device. (ii) Offers a discrete undergarment opportunity that can be designed so as not to convey a medical condition in everyday life events. i.e. school environments, school trips. This would be important for any future use of the device for other indications than NE. Several fit ranges have been developed to accommodate various population morphological types.

**Table 4:** Waist and upper thigh circumferences based on the age groups [24]

	Male					Female				
Age	*N*	Mean	sd	Min	Max	*N*	Mean	sd	Min	Max
Upper thigh circumference (cm)										
6.5–7.5	121	35.6	3.6	32	39.2	102	37.1	4.1	33.0	41.2
7.5–8.5	94	37.5	4.3	33.2	41.8	95	39.4	4.6	34.8	44.0
8.5–9.5	137	39.2	4.0	35.2	43.2	111	41.1	4.8	36.3	45.9
Waist circumference (cm) on the navel										
6.5–7.5	104	54.3	5	49.3	59.3	120	55.3	6.3	49.0	61.6
7.5–8.5	96	56.6	5.7	50.9	62.3	93	57.0	6.5	50.5	63.5
8.5–9.5	114	58.3	5.9	52.4	64.2	137	59.2	7.0	52.2	66.2

**Table 5:** Measured values on the hypo-gastric region with respect to pubic bone. Reading (2/3–10 mm) corresponds to the reading 10 ml below the centre of the gel-pad whereas Reading (2/3 + 10 mm) corresponds to the reading 10 cm above the centre of the gel-pad

Volunteers	Age	Dimension	Pubic-reading	Reading	Reading	Height	Weight	Gender	Waist	Thigh	BMI
		A-B (cm)	(cm)	(2/3–10 mm)	(2/3 + 10 mm)	(cm)	(kg)		(cm)	(cm)	
MP1	7	13.5	4.5	✓	✓	126.5	26.5	F	52	31	18.6
MP5	8	11.3	4.4	✓	✓	129.5	26.7	F	66	42	18.2
MP6	9	9.4	3.6	X	✓	134.5	20.1	F	59.5	39	12.3
MP7	9	15.8	5	✓	✓	148	48	M	85	51	23.5
MP8	7	8	2.5	✓	✓	127	25	M	58	35	17.9
MP9	9	13	4.5	−	−	137.2	26	M	53.5	39	15.4

### Comfort trials

The undergarment, along with the space model and the gel pad as presented in Fig. 14, has been tested with 5 non-NE children for 1 week. The self-adhesive skin-interfacing gel pad that is sticky on both sides and used to keep the sensors fixed to the abdomen in order to remove the acoustic coupling risk is also shown in Fig. 14c. The families and children have completed a questionnaire at the end of the trials under the supervision of two psychologists. Interested readers can find the questionnaire in the supplementary materials. This undergarment together with the space model and the gel pad has been found to be quite comfortable based on the feedback and the results obtained from those questionnaires.

### Hardware design

The reflection of the pulses from the anterior wall of the bladder where the bladder is empty is 99.9% [27], which illustrates the difficulty in transmitting emitted pulses beyond the distal side of the bladder. It is 5% [27] where there is urine by which an emitted signal can reach the posterior wall without any difficulty through the urine. The characteristics of the bladder along with its environment change according to the sex, age, body mass index (BMI), amount of urine inside, and position of the body. Therefore, there is no specific definition of the bladder shape and a customisable device that incorporates a proper design of sensors is required to be able to acquire echoed pulses from the bladder regarding the reflections and refractions of US beams as explained in our previous paper [1] specific to the characteristics of the user. The sensor design in the MyPAD project is explained in Section 2.6.1.

An ergonomic wireless US device has been developed by our collaborator, NovoSound Ltd. Integration of all required components in an appropriate device case is being carried out by the University of Central Lancashire. Other sensors such as temperature, moisture detectors, and movement measurement for determining postural changes are being incorporated into the device to enhance warning performance and self-customising features. The main components of the MyPAD device are explored in Sections 2.6.1, 2.6.2, 2.7.1, and 2.7.2.

**Fig. 15:**
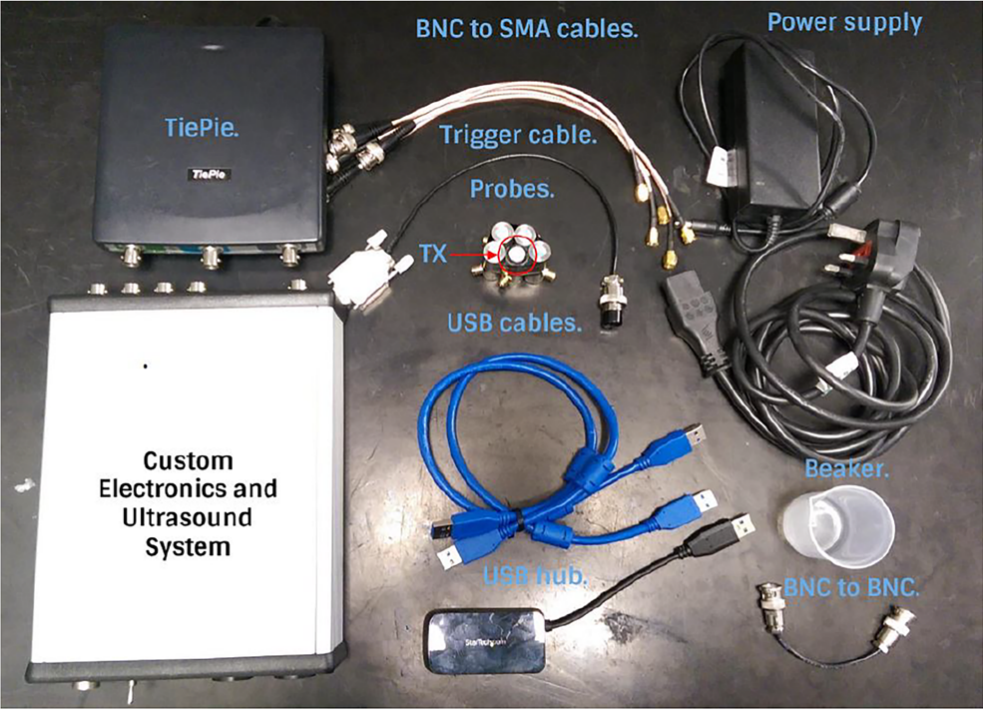
The components of the bespoke device: 1 TiePie, 1 custom electronics and US system, 5 BNC to SMA cables, 1 trigger cable, 1 BNC to BNC cable, 2 USB cables, 1 USB hub, 1 power supply, 4 receivers (EX) and 1 transmitter (Tx)

#### Development of the custom electronics and US system

**Fig. 16:**
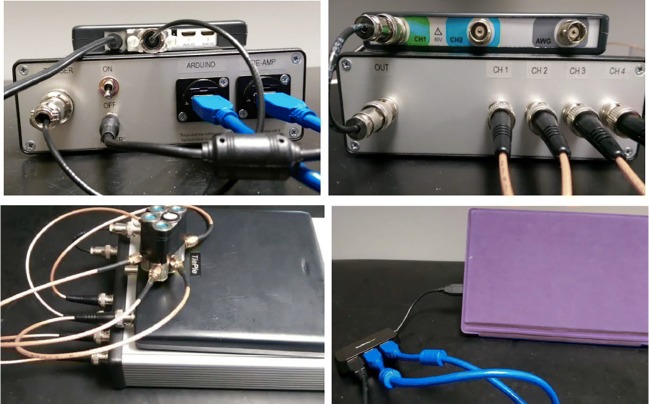
Connection of the system components

**Fig. 17:**
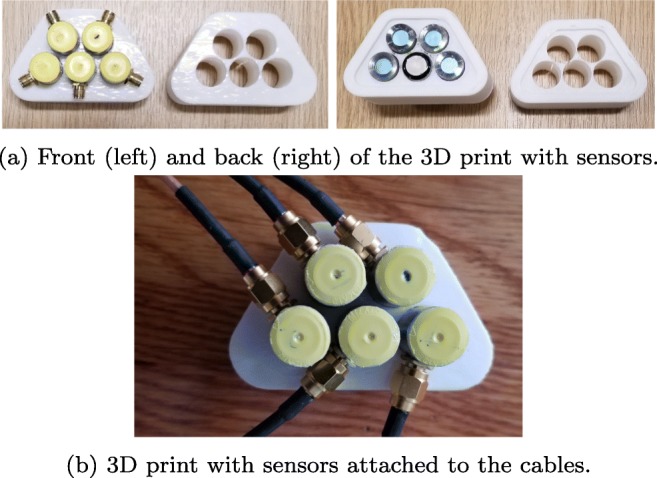
Sensors placed in the 3D print. Each probe could also be at a different angle, essentially focusing them to different depths

The components of the bespoke electronic device are presented in Fig. 15. These components are 1 custom electronics and US system, 1 TiePie oscilloscope,^2^ 5 BNC to SMA cables, 1 trigger cable, 1 BNC to BNC cable, 2 USB cables, 1 USB hub, 1 power supply, 4 receivers (EX), and 1 transmitter. The connection of these system components is shown in Fig. 16. The properties and functions of the hardware components are explained in Table 6. The high-efficiency piezoelectric micromachined ultrasonic transducers (PMUT) connected to programmable system-on-chip (SoC) unit have been developed using micromachined piezoelectric crystals. Those crystals are being housed and protected by a cylinder-shape metal case in order to make the preliminary test effectively without damaging the crystals and the connection of the crystals with the electronics as shown in Fig. 17 a and b in which the design of a group of transducers is displayed as well. Plurality of the sensors is incorporated into MyPAD device design to detect the echoed pulses reflecting from the bladder and its surrounding media regarding the various refraction properties that are explained in [1].

**Table 6:**
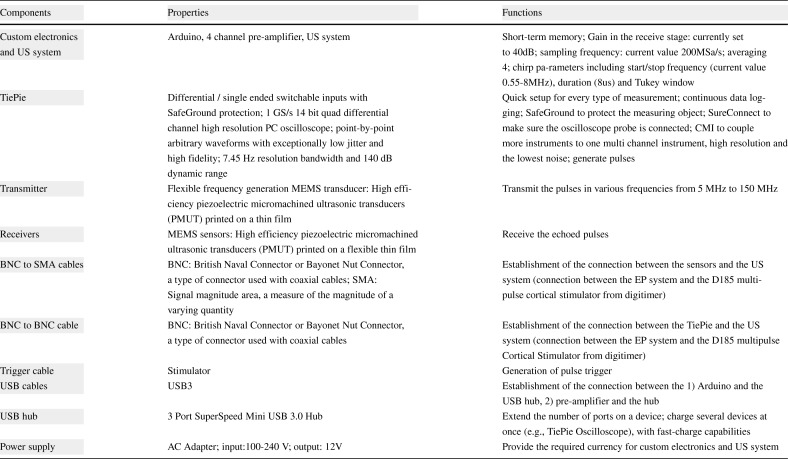
Features of the custom electronics and US system in the bespoke hardware design presented in Fig. 16

**Fig. 18:**
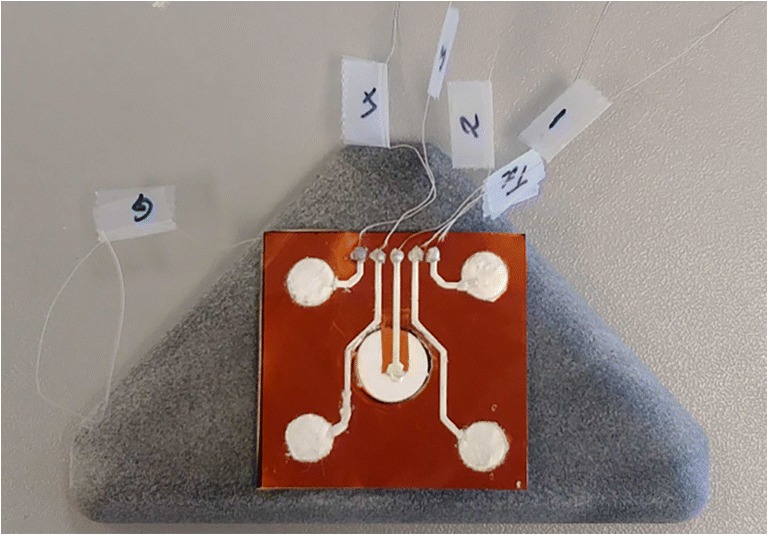
The design of the five probes printed on a flexible film based on the space model

**Fig. 19:**
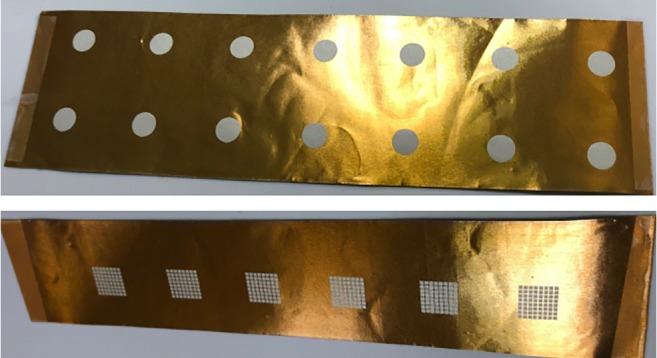
Flexible films on which sensors are printed

The piezoelectric micromachined ultrasonic crystals are being printed on the flexible thin films by our collaborator NovoSound Ltd in order to make the transducers ergonomic as shown in Fig. 18 involving the outer protective film as depicted in Fig. 19. This film mainly protects the sensors from outer impacts and absorbs the noise created by the US during pulse generation. This design is expected to increase the satisfaction of the users regarding more comfortable and ergonomic use of the device when compared with the space model used during the trials explored in Section 2.5. There is one pulse generator as a beam transmitter (the white component in Fig. 17a) to emit the pulses and 4 receivers (the blue components in Fig. 17a) to acquire the echoed pulses. In this way, echoed pulses are aimed to be acquired using at least one of the sensors based on the properties of the US refraction and reflection physics. Additionally, the principles of ALARA (As Low As Reasonably Achievable) are pursued by emitting pulses from only one generator rather than five generators.

We are currently testing MEMS sensors with higher frequencies (e.g., 5–10 MHz) that increase the resolution, but, 2nd harmonic cannot be detected regarding the doubling frequency (e.g., 10–20 MHz) due to strong attenuation in the human body as explained in our previous study [1] in detail. Therefore, development of similar transducers with lower frequencies is underway in order to use non-linear crisps particularly for the harmonics (e.g. 2nd harmonic) generated in the urine, which was explained in the supplementary materials of our preliminary study [1] where we were using sensors in low frequencies (e.g. 2.2 MHz). We would like to employ non-linear crisp on harmonics in the urine when we have the MEMS sensors in lower frequencies, and we plan to cover these issues with more details presenting the latest design of the MEMS sensors in our following paper involving planned comprehensive tests on children as milestones.

#### Development of the bed-side alarm box

**Fig. 20:**
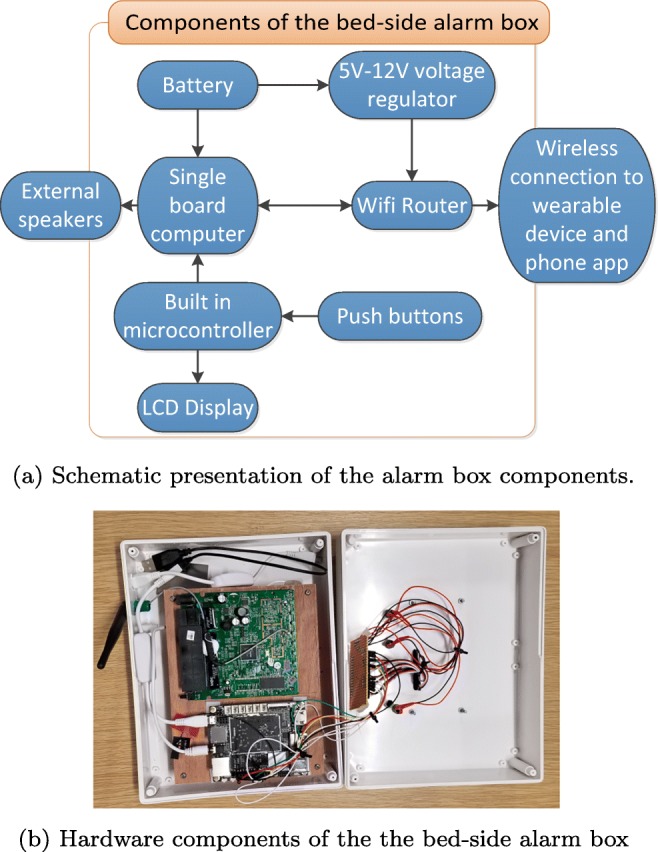
Components of the the bed-side alarm box

Integration of all required components in an appropriate device case is being carried out by the University of Central Lancashire. The components of the bed-side alarm box in Fig. 2 are depicted in Fig. 20 a and b. The alarm box single board computer is a Lattepanda developed by DFRobot. This was chosen as it is a highly capable board with a 1.8 GHz quadcore processor, 2 GB of DDR3 RAM, and built-in micro SD card slot. The Lattepanda also has a built in microcontroller that can be programmed using the Arduino IDE. This enables the board to easily interface with the push buttons and the LCD display. Within the alarm box is a simple WiFi router to provide reliable communications with the MyPAD device without having to rely on the user’s own wifi network. A battery is also included within the box to allow the alarm to operate for short periods without a main connection.

Other sensors such as temperature, moisture detectors, and movement measurement (i.e. angular accelerometer) for determining postural changes are being incorporated into the further development of the MyPAD device to enhance the warning performance and self-customising features. The bed-side alarm box has been built to work stand-alone to analyse the echoed pulses and trigger desired alarms without needing a cell phone as illustrated in Fig. 1, which is also a request of the children with NE and their families as concluded in Section 2.2 during the workshop.

### Software design

The Agile SW development approach has been employed in the application development phase.

#### Development of the data acquisition interface

**Fig. 21:**
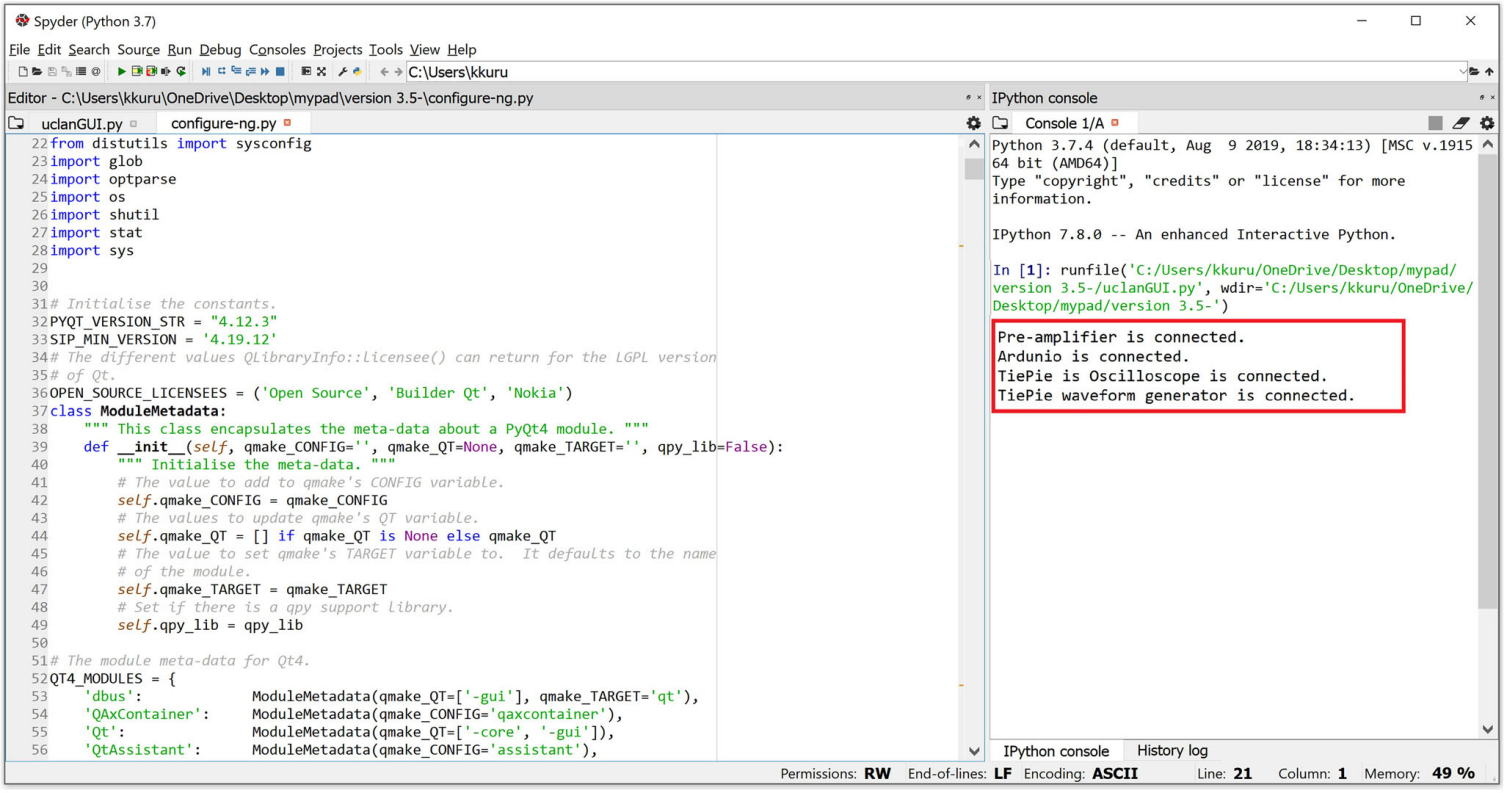
The data acquisition interface

The run-time programming interface used by the SW engineers is depicted in Fig. 21. The bespoke interface has been developed using Python programming language within Anaconda Spyder platform to orchestrate the components of the MyPAD device, propagation of pulses, and detection of the echoed pulses. The re-amplifier, Ardunio and TiePie oscilloscope, explained in Section 2.6 is connected all together when the application is run. The application has been developed based on the properties of the physics of US with respect to the characteristics of the human body, particularly the bladder and its surrounding media. The expansion of the bladder regarding the height and width shows a similar pattern. Our recent tests on the 7–8 age group that is explained in Section 2.3 have shown that the maximum width or height of the bladder is 9 cm and the maximum distance of the bladder from the abdomen is 2 cm where the bladder is full. The targeted region of interest (ROI) is specified as 15 cm maximum for this age group in order to ensure that all related area is covered.

**Fig. 22:**
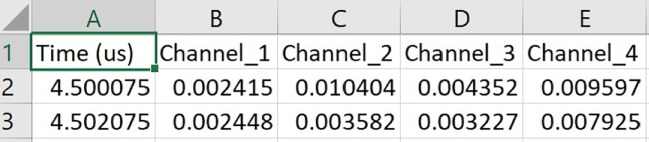
The data saved in the CSV format: First column is the time in nanosecond (ns) and the other four columns are the channel outputs in Volts

The filling speed of the bladder is slow during the night while sleeping because there is no further fluid intake, and depending on maturity, there is greater production of antidiuretic hormone with consequent reduction in urine production. One hundred-milliliter urine changes the bladder wall thickness 3 mm (from 5 to 2 mm) from 150- to 250-ml urine, which helps trigger the voiding need [28]. This filling speed in children is between 10 ml/min and 20 ml/min at medium-fill cystometry whereas slow-fill cystometry (’physiological filling’) is 10 ml/min [29]. Therefore, 2 min for taking measurement using 3 consecutive US pulses is selected in order both not to miss any possible triggering point of voiding need, and ensure that the exposure time and intensity of US applied are the minimum regarding the ALARA principles. Additionally, in this way, the power of the battery used in the MyPAD device will be saved. The acquired echoed pulses are saved in a CSV format as depicted in Fig. 22. The A-mode data acquisition interface of the MyPAD device is explored in Section 2.8 regarding the tests on phantoms and volunteers.

#### Application development of the smartphone and bed-side alarm box

**Fig. 23:**
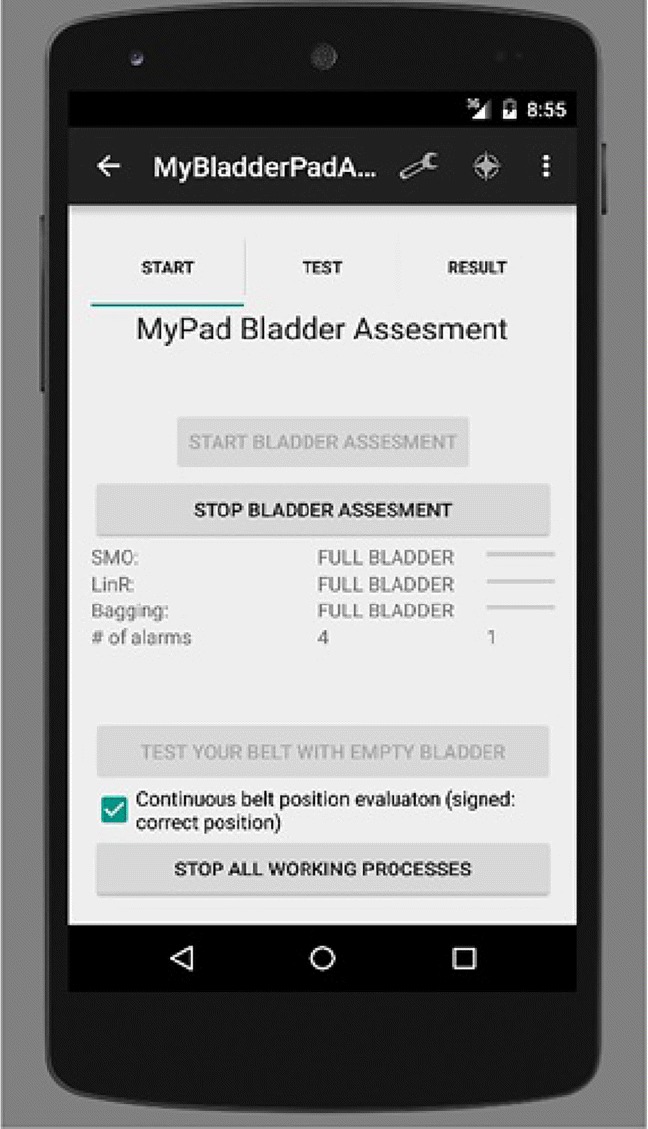
Determination of bladder status and control of the undergarment placement

A smartphone application was built using Java programming language within Android Studio platform to analyse the distinctive features of the echoed pulses generated from the urinary bladder, urine, and surrounding media. The main interface that the user communicate with the system is presented in Fig. 23. The other interfaces of the smartphone ML algorithms were presented in our previous study [1] in detail.^3^ Similarly, an application was built using C Sharp (C#) programming language to work on the Windows operating system for the bed-side alarm box to trigger alarms at the desired time (Fig. 20b using a small and simple interface using an LCD screen (as shown in the red frame in Fig. 2). The first two buttons above the LCD screen from left to the right, namely red and yellow ones, are currently active. The red button is used for the feedback from the user to indicate that a false alarm is triggered by the system in addition to silencing the alarm whereas the yellow button is used just to silence the true alarm. The other two buttons are designed to be associated with the fluid intake and voiding activities in the further development of the system. To summarise, the application analyses the acquired echoed pulses with respect to the pre-trained models (e.g. voiding need model) and comes up with a decision such as empty bladder, half bladder, or voiding need for triggering an alarm. More explicitly, the application employs three ML techniques along with RL on the dataset using holdout, *n*-fold, and leave-one-out cross-validation (CV) schemes depending on the number of the instances. The ML techniques that fit the datasets best are sequential minimal optimisation (SMO), linear regression (LR), and ensemble bagging (EB) meta-learning algorithms that were tested on sample datasets and found successful regarding the sensitivity (*Se*) and specificity (*Sp*) values. Final bladder status is determined by the weighted average/majority of the plurality of three distinct bladder status opinions as new instances are introduced into the system, and accordingly, a pre-void alert type customised for the user (audible and/or vibrating alarm) is triggered if the level is indicating a triggering point. The alert signal may also be received by a third party’s (e.g. parent or carer) smartphone optionally to inform them to check the child.

The application will come to the market with general trained models specific to age, sex groups, and morphology types and has the ability to be customised for the user. It is supported with RL abilities to enable self-learning, self-optimisation, and self-configuration functions by using the autonomous feedback as mentioned in our previous paper [1]. More explicitly, the device has been designed to record a voiding event through the device’s moisture sensor detecting wetness in the case of an involuntary voiding event to customise itself for the current user in terms of the triggering threshold using self-tuning features as illustrated in the scenarios in [1]. Additionally, the user feedback is prime important using the red button on the alarm box (Fig. 2) where a false alarm is triggered by the system causing sleep interruption with no reason. The system adjusts its training abilities based on these inputs and sets a customised warning trigger point based on the bladder expansion cycle with respect to the likelihood of an imminent voiding of the bladder by learning the bladder characteristics specific to the child.

### Test of the device

#### Tests on the phantom

**Fig. 24:**
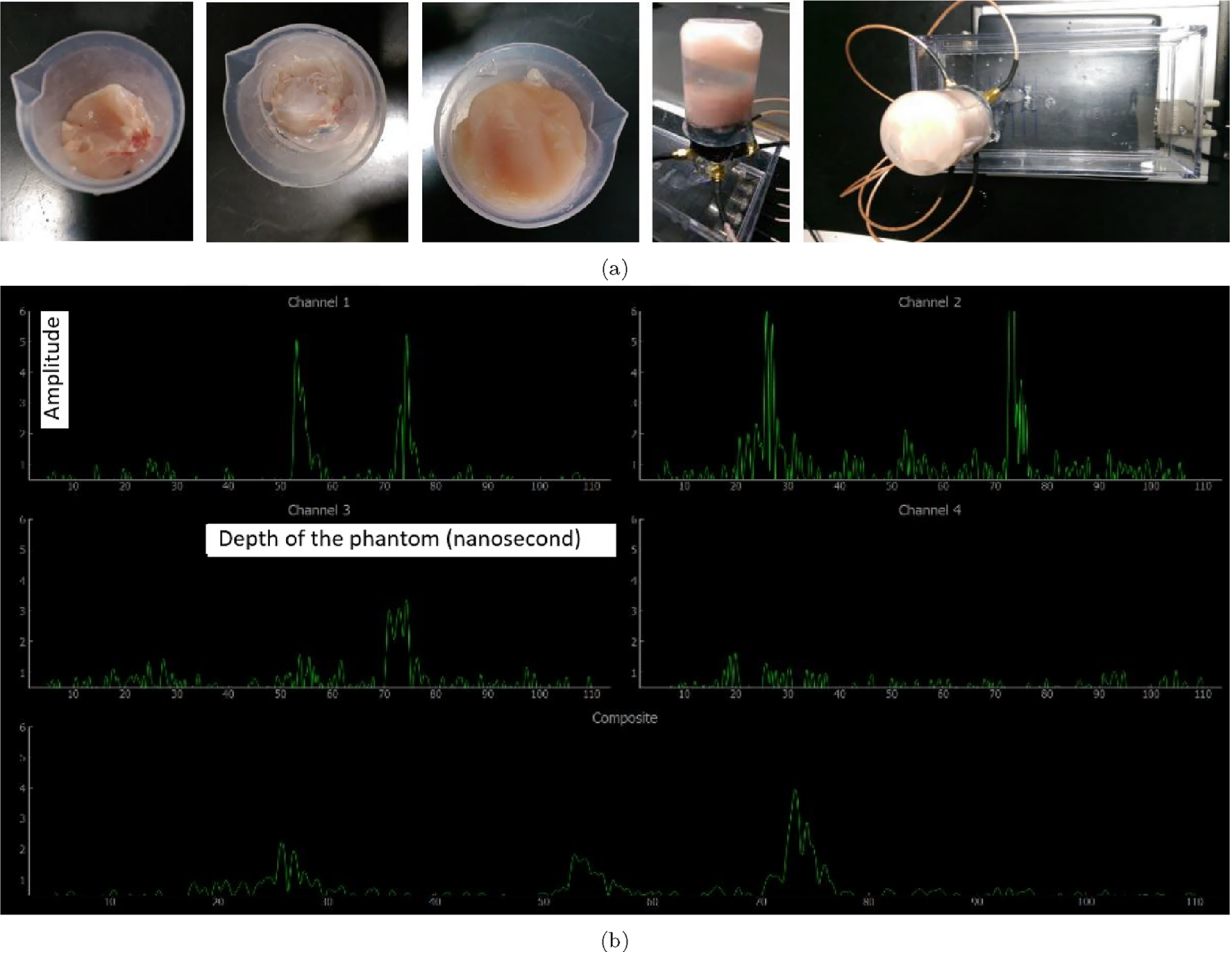
**a** Phantom and the test design of sensors on the phantom, and **b** each channel corresponds to the echoed pulses acquired from each probe. The composite graph is an averaged signal form all four channels

A phantom that imitates the characteristics of the human bladder and its surrounding media was developed to test the device in order to improve its detecting ability before applying the device to volunteers as presented in Fig. 24a. First, a small layer of chicken tissue was put into the bottom of a beaker. Second, a required amount of US gel was poured on the chicken layer placed at the bottom. Third, another layer of chicken tissue was placed at the top of the beaker on the US gel. No air-bubble was left in the gel placed between two chicken layers in order to both ensure the beams propagate to the posterior chicken layer through the gel and desired results are acquired with the output readings. An amount of gel was poured on the chicken layer at the top of the beaker before placing the sensors on the phantom. The SW was improved through several iterations and the final output observed with the phantom data is displayed with the data acquisition interface in Fig. 24b. The four sensors are used to detect the echoed pulses generated with the pulse generator and the acquired signals from each sensor are recorded using four channels in a multi-thread processing way by which the tasks related to the pulse generation, detection, and processing of echoed pulses are performed in parallel at a time. The acquired echoed pulses for sensors are shown separately in the dedicated sections labelled as ‘Channel 1’, ‘Channel 2’, ‘Channel 3’, and ‘Channel 4’ . Those four channels are processed with respect to the amplitude and time (i.e. depth) to form a composite representation in the dedicated section labelled as ‘Composite’. All the amplitudes at the same depth in these channels are summed and divided by four to find the final composite amplitudes. In this way, the nearest part and the furthest part of the bladder can be determined, and accordingly, the volume of the urine can be measured with respect to the biggest expansion of the bladder based on the specific characteristics of the user.

#### Tests on the volunteer

**Fig. 25:**
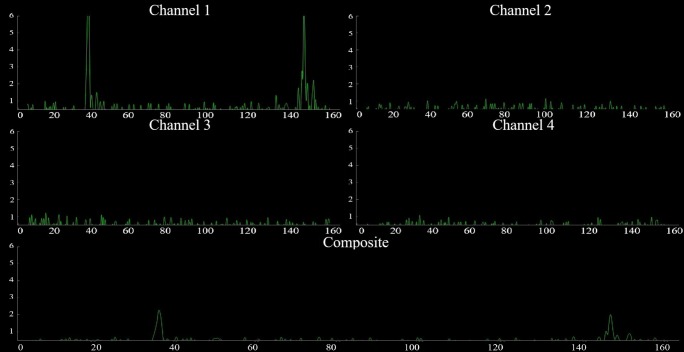
Echoed pulses acquired from the full bladder and their composite presentation

**Fig. 26:**
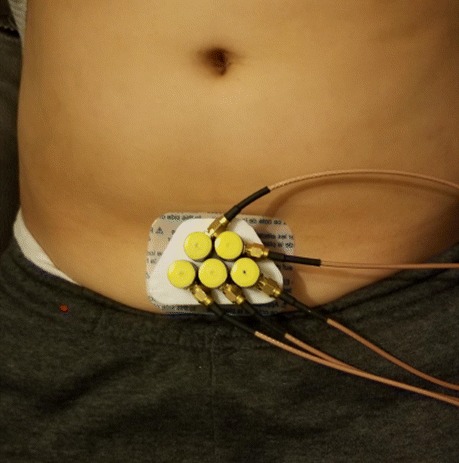
Sensors placed in the 3D print acquiring echoed pulses from the bladder

The 3D print (Fig. 17b) is used to place the pulse transmitter and receivers. The device was tested on a 9-year-old male child and the resultant screen-shot of the echoed pulses acquired from the full bladder using one sensor and its representation in the first channel and the composite form is shown in Fig. 25 in the supine position as displayed in Fig. 26 involving the transmitter and receivers housed by the 3D print. The first amplitude at 35 ms indicates the anterior wall of the bladder whereas the second amplitude at 145 ms corresponds to the posterior wall.

## Results and discussion

Development of a safe, comfortable, and non-invasive pre-void wearable alarm and associated technology using advanced mechatronics to treat bedwetting is the main objective of this study. In this manner, this study was carried out to explore whether existing technologies could be synchronised, enhanced, and modulated to form an intelligent alarm system with AMS components that could provide a pre-void warning, minimising bedwetting, reaching stable dryness through learning bladder control and enhancing quality of life for children who wet the bed. The results suggest that the samples acquired from the a single element US device and conventional US device correspond to the same status of the bladder. The maximum ROI for the pulse generator and the sensors should be 15 cm for 7–9-year-old children to ensure that all related area is covered. During the tests, the children consumed liquid during the data acquisition phases in order to both observe the characteristics of the expansion with respect to the consumed liquid and reduce the total test time to be able to observe scenario with the fastest filling of the bladder. The posterior wall can be detected between the second trial and third trial, in around 30 min where the expansion of the bladder starts above the 50-ml urine volume and the expansion increases almost in a linear form. The bladder fills in 100 min in four of the children when they were drinking as much as possible. The voiding need starts in approximately 65 min (i.e. 2/3 full bladder status) for 7–9-year-old children and 80 min for 14-year-old children that can change slightly from person to person regarding the bladder size and the specific characteristics of individuals. Please note that the children coded as MP3 and MP4 ended the trials earlier since their full bladder measurements are significantly lower than the normal ranges as explained in Section 2.3. Bladder volumes vary between individuals and have large standard deviations when working on the dynamic environment of the bladder as presented in Table 2. Therefore, the techniques employed in this study are designed to customise the MyPAD device for the specific characteristics of individuals through continuous learning supported by RL techniques. More explicitly, the techniques are developed to analyse and learn the patterns of all acquired echoed pulses within a dedicated ROI (e.g. 15 cm for 7–9-year-old and 20 cm for 14-year-old children) with respect to the specific features of the individuals rather than calculating the urine volume, in particular, patterns of echoed pulses that correspond to the voiding need. These patterns were found successful to discern the bladder volume status of empty, 1/2 (half), 3/4 (three quarters), and full [1]. Further tests on more children using recently developed MEMS sensors printed on a flexible thin film (Figs. 18 and 19) are required to determine if the patterns indicate a specific volume of urine, which is planned and explained in Section 6. We are keen to perform this process in comparison with the voided urine of individuals since the conventional US modality is an approximation of the real volume,^4^ and the gold standard catheterisation is invasive, uncomfortable, and introduces the risk of infection and trauma as explained in Section 1. *Se* observed using the conventional sensors and gel as mentioned in our previous paper [1] was 0.89 resulting in 11 false alarm out of 100 and causing sleep interruption with no reason. We are targeting to achieve a *Se* value of over 0.95 with the new design explored throughout this paper, which is intended to be improved with autonomous feedback and child input as the device is used.

Regarding the workshop mentioned in Section 2.2 , (1) The families are desperate about the current moisture alarms and medicine they are using, (2) all these 4 children are suffering during day-time as well and they need to visit the toilet regularly to avoid any involuntary daytime voiding, (3) they would prefer using a pre-void alarm system, (4) The prominent features of such a system emphasised by the families and children are comfortable design and easy-to-use abilities regarding the hardware, SW, and undergarment, and most importantly (5) The children do not want to be different from their peers in appearance while using the device.

The solution for treating the bedwetting in this study is to design and develop an intelligent autonomous AMS to trigger a pre-void warning that can be customised to the user’s specific physical characteristics by combining several measurement attributes of the bladder when it is full, expanded, or empty. In this manner, we aim to deliver a compact AMS device with easy-to-use interfaces in a compact package that can be used by children without needing any engineer or parent. This device will be unique in that it recognises the warning signs of a pending emptying of the bladder via tracking expansion of the bladder volume over time, and will wake the patient up in time to prevent it. This process is customised or tuned to an individual patient’s bladder volume trigger point. This more accurate advanced warning system will help the children to alter their behaviour over time, reducing the frequency of NE [30] through learning bladder control over time. The main advantage of our techniques using single element MEMS sensors is its simplicity, safe, low cost, and most importantly comfortable use. In the long run, larger collection of data samples from different sexes, age groups, and morphology types using the cloud platform as disclosed in Section 6 can be processed to improve the performance of the MyPAD device and train a stand-alone system that can be employed for larger range of NE patients with a very short customisation period.

Beyond this study, there are numerous other areas of application i.e. elder care (geriatric) settings, stroke patients [31], diagnosis of urinary retention, and veterinary science in which My-PAD can be of potential benefit.

## Limitations of the study

We have studied a small number of children and this has been sufficient to guide the development of the device, but recruiting more children in subsequent phases of the project will help getting more data on bladder size and voiding points based on various morphology types. Additionally, the results obtained from a single element US are compared with the approximate measurements in the images obtained from conventional US device until the voided urine is finally obtained and measured. We would like to note that it would be better to compare the results with the measurements acquired from the gold standard catheterisation for safer conclusions. However, we could not use catheterisation, because, it is invasive, uncomfortable to the patient, and introduces the risk of infection and trauma as explained in Section 1.

## Conclusion

The approach being taken with this study is to develop an external and non-invasive wearable bladder monitoring system that will wake the child up when a possible urinary voiding event is predicted rather than merely responding to the presence of moisture after an event has taken place as conducted by the current post-void alarm devices in the market. More particularly, the present study relates to methods and apparatuses for treating urinary incontinence, suitably by providing pre-void alerts that allow a patient to void in a dignified manner. The components of the MyPAD device built in this study have been tested successfully both on the phantoms and volunteers.

## Future directions

**Fig. 27:**
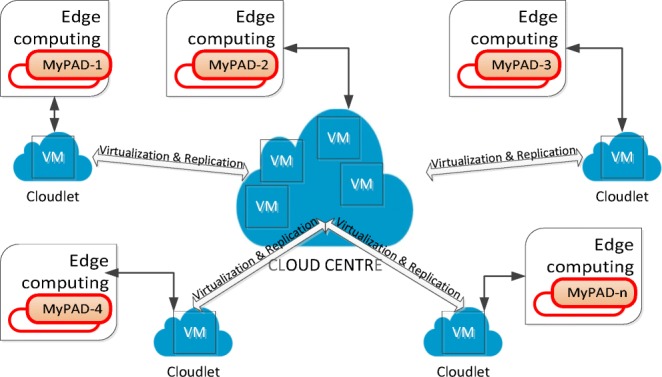
MyPAD AMS within the edge and cloud platforms

A miniaturised version of the composite system explained in this study is being developed. Additionally, in the new version, the cables will be removed and the communication, in other words, data transformation will be performed via a wireless communication way from sensors to the device, which will also make the device more comfortable during sleep. A total of 10 units of this miniaturised version are going to be developed for the children with NE to be used for 14 weeks after testing the first miniaturised version successfully. The MyPAD devices with AMS abilities, more explicitly, location independent monitor and control abilities will be able to work within the edge and cloud platforms as illustrated in Fig. 27 using a miniaturised single-board computing component. The information about the performance of the device and collected data will be saved in the cloud platform to be analysed further and to improve the performance of the device in the long run. In this regard, the MyPAD devices used by the children will have the location independent monitoring and controlling abilities using the sanitised data within the consent of the children and their families, that is, privacy and security concerns will be prioritised. Additionally, we are recruiting more children to be able to tailor the most suitable garment for various morphology types and age groups by incorporating the outcomes of the study [24] mentioned in Section 2.4. The application is planned to be enhanced with the code development standards of ISO/IEC/IEEE 12207:2017 during the commercialising phase.

## Electronic supplementary material

Below is the link to the electronic supplementary material.

(PDF 2.96 MB)

## Footnotes

1 Interested readers can find a comprehensive analysis of these products and medications in our previous study [1].

2 Interested readers can find more information about TiePie oscilloscope on https://www.tiepie.com/en.

3 The motivated readers are referred to the study [1] for detailed information about the techniques employed and the interfaces built.

4 The values are not strictly consistent within the measurements of the conventional US device: The value of wall thickness is increasing after 2nd trial for MP6; the value of height is decreasing at the 3rd trial for MP6; the value of skin and bladder distance is increasing at the 3rd trial for MP1.
