# Supplementary material for: Intelligent autonomous treatment of bedwetting using non-invasive wearable advanced mechatronics systems and MEMS sensors: Intelligent autonomous bladder monitoring to treat NE
Source: Med Biol Eng Comput. 2020 Feb 24;58(5):943–65. doi: 10.1007/s11517-019-02091-x (PMC7188739; doi:10.1007/s11517-019-02091-x)
Supplement: Supplementary file 1 — (PDF 2.96 MB) [file 11517_2019_2091_MOESM1_ESM.pdf]

Noname manuscript No.
(will be inserted by the editor)

Intelligent autonomous treatment of bedwetting using non-invasive wearable advanced mechatronics systems and MEMS sensors -Supplementary Materials-

Kaya Kuru · Darren Ansell · Martin Jones · Benjamin Jon Watkinson · Noreen Caswell · Peter Leather · Andrew Lancaster · Paula Sugden · Eleanor Briggs · Carl Davies · Chooi Oh · Kina Bennett · and Christian De Goede

Received: date / Accepted: date

1 Bladder expansion

Financial support for this study was provided entirely by a grant from the National Institute for Health Research (Invention for Innovation, i4i; MyPad - Intelligent Bladder Pre-void Alerting System; II-LA-1116-20007). The funding agreement ensured the authors' independence in designing the study, interpreting the data, writing, and publishing the report.

K. Kuru
University of Central Lancashire, School of Engineering, Fylde Rd, Preston, PR1 2HE, UK.
Tel.: +44-1772895156, E-mail: kkuru@uclan.ac.uk

D. Ansell
University of Central Lancashire,

M. Jones, B. J. Watkinson, N. Caswell and P. Leather
University of Central Lancashire,

A. Lancaster and P. Sugden
Lancashire Teaching Hospitals NHS Foundation Trust,

E. Briggs and C. Davies
University of Central Lancashire,

C. Oh, K. Bennett and C. DeGoede
Lancashire Teaching Hospitals NHS Foundation Trust,

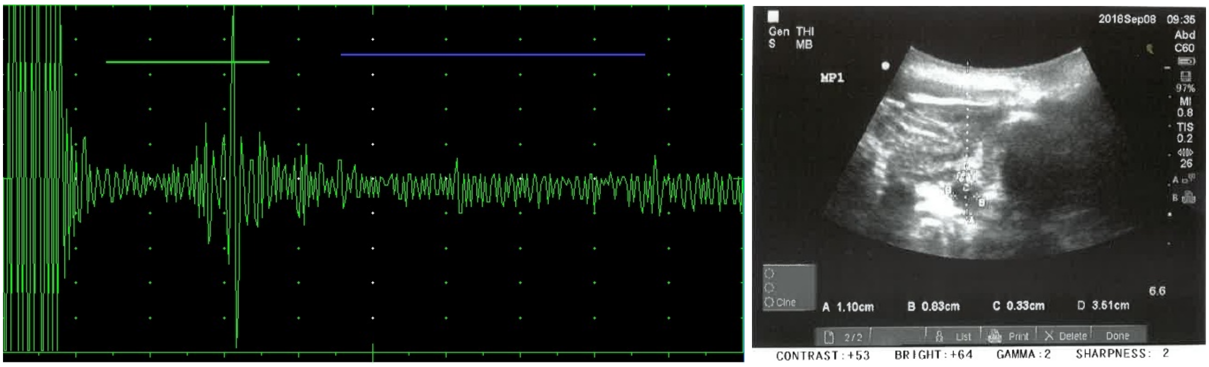

Fig. 1: Volunteer MP1.

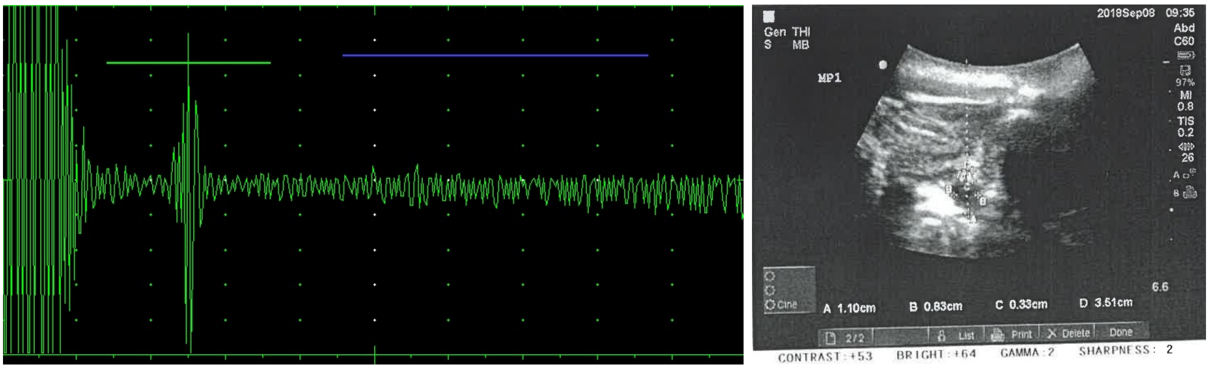

Fig. 2: Volunteer MP1.

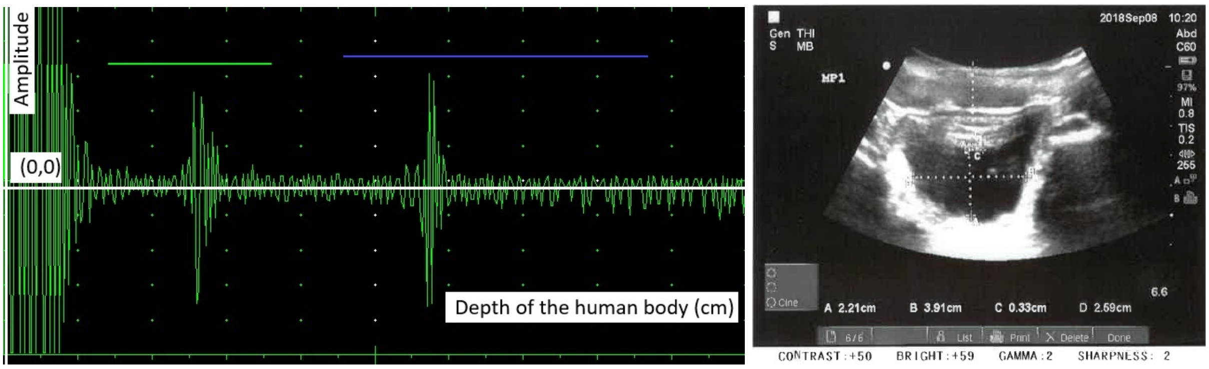

Fig. 3: Volunteer MP1.

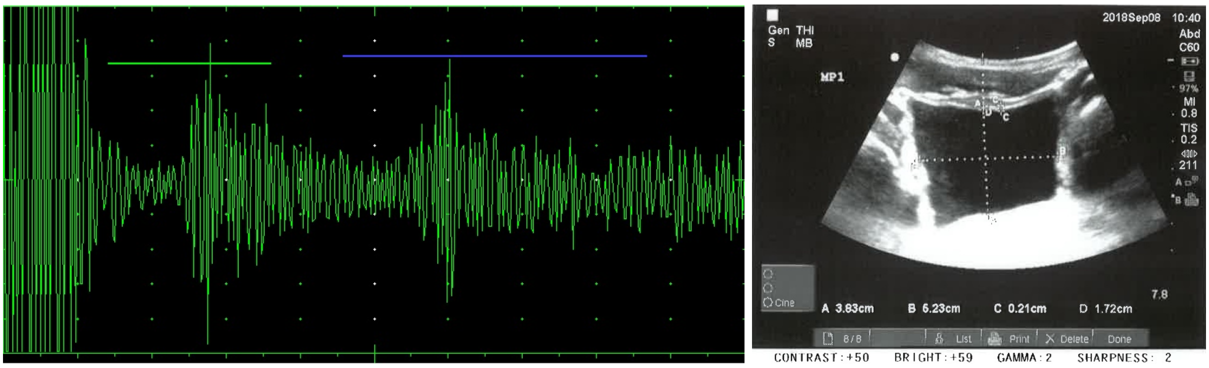

Fig. 4: Volunteer MP1.

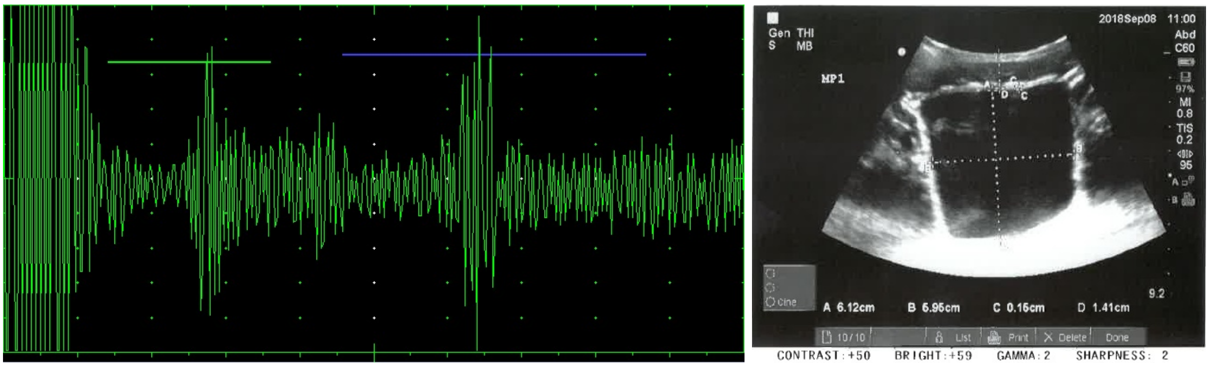

Fig. 5: Volunteer MP1.

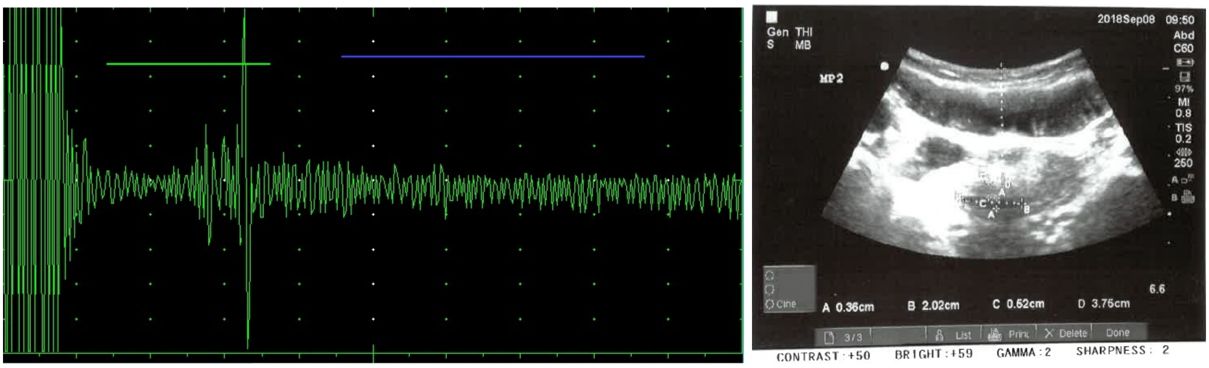

Fig. 6: Volunteer MP2.

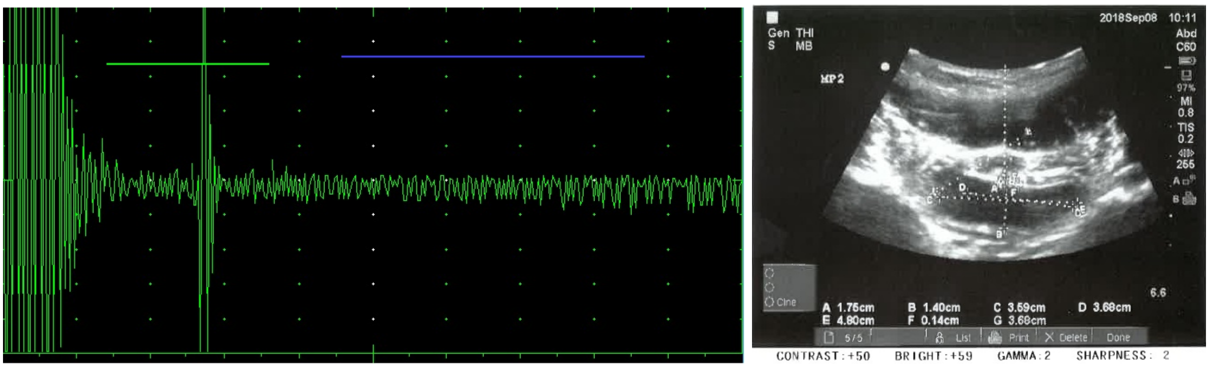

Fig. 7: Volunteer MP2.

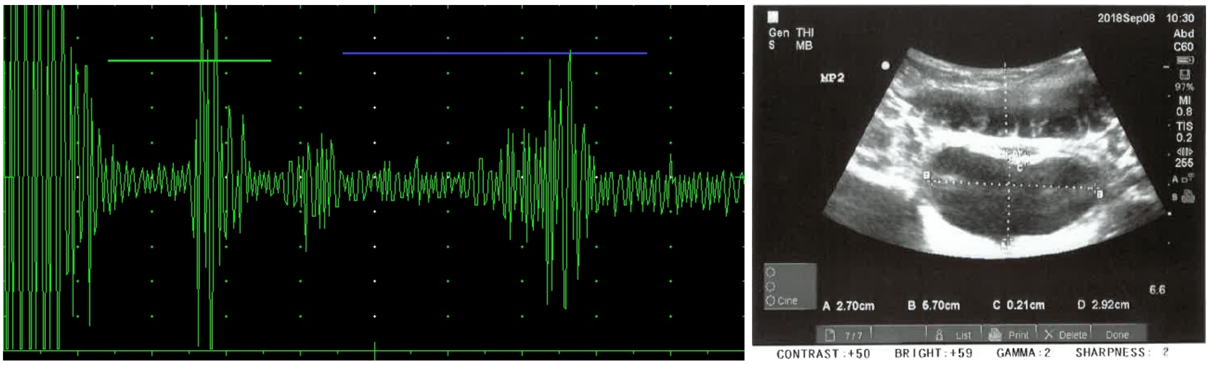

Fig. 8: Volunteer MP2.

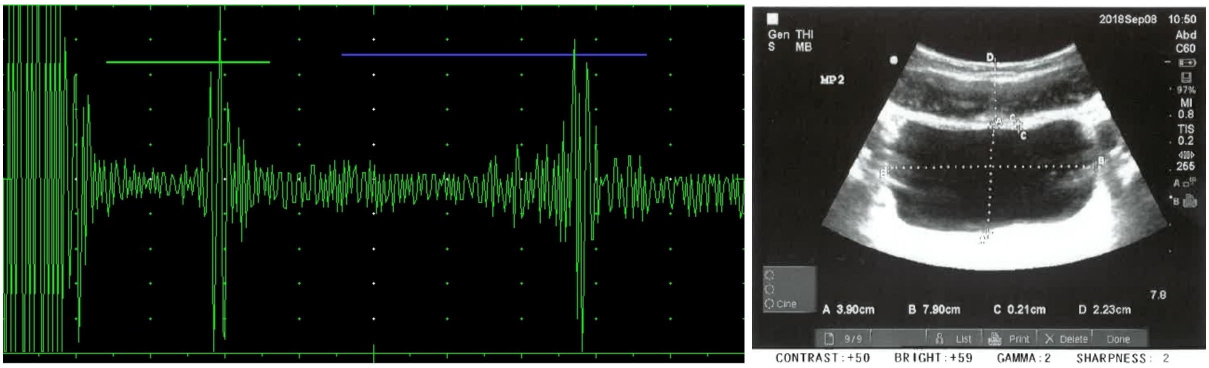

Fig. 9: Volunteer MP2.

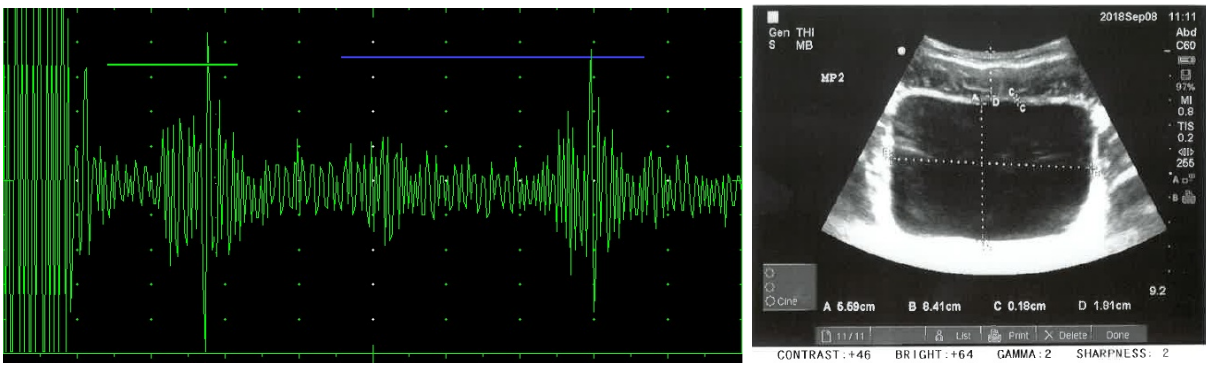

Fig. 10: Volunteer MP2.

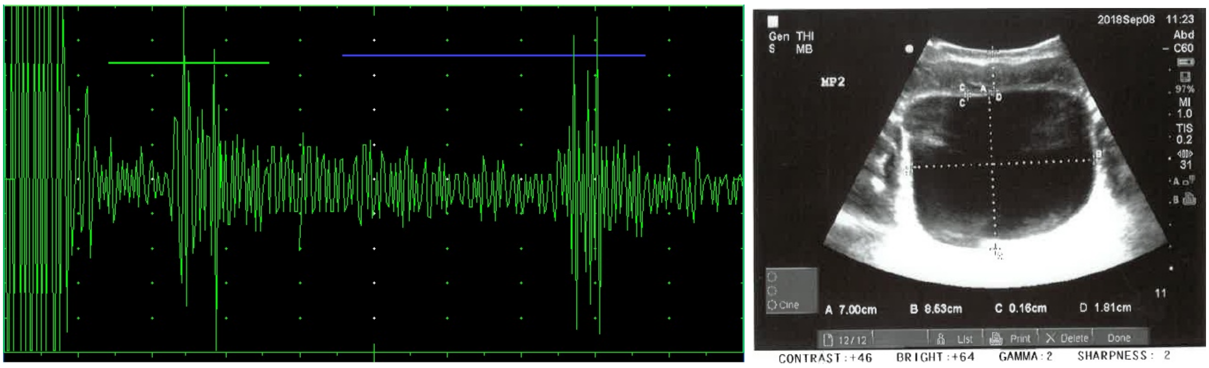

Fig. 11: Volunteer MP2.

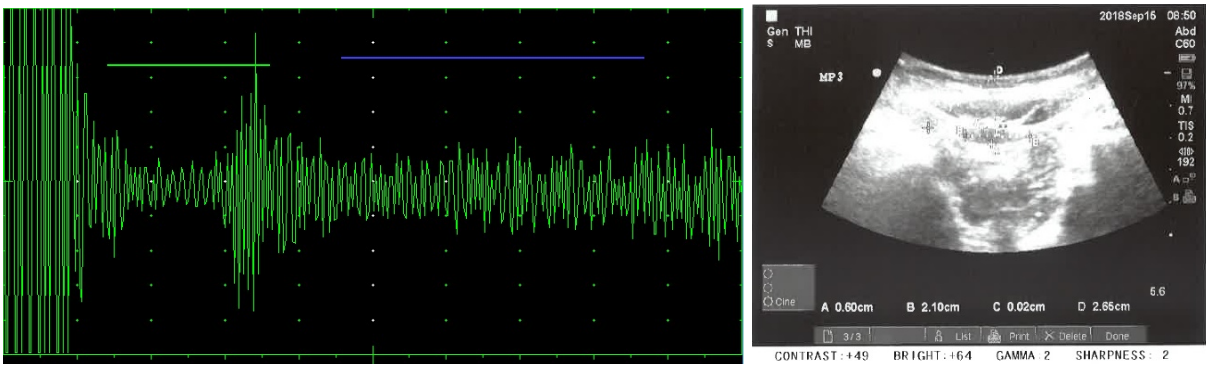

Fig. 12: Volunteer MP3.

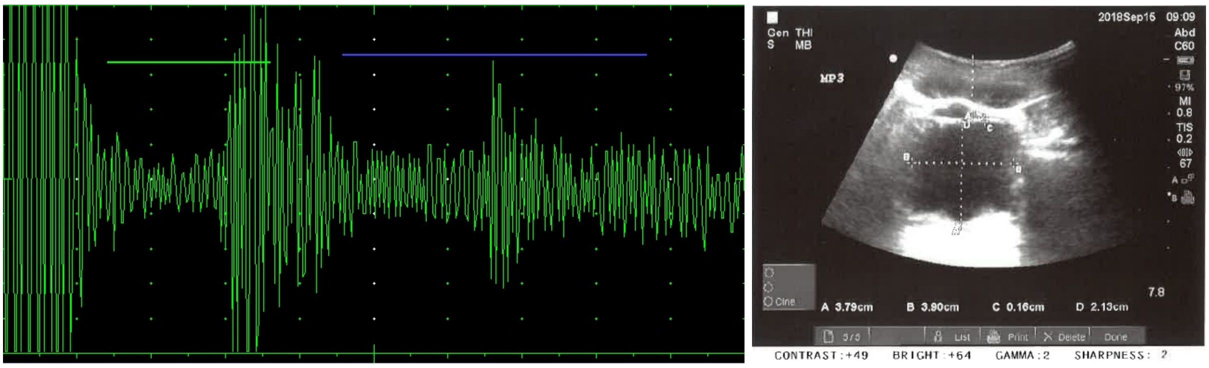

Fig. 13: Volunteer MP3.

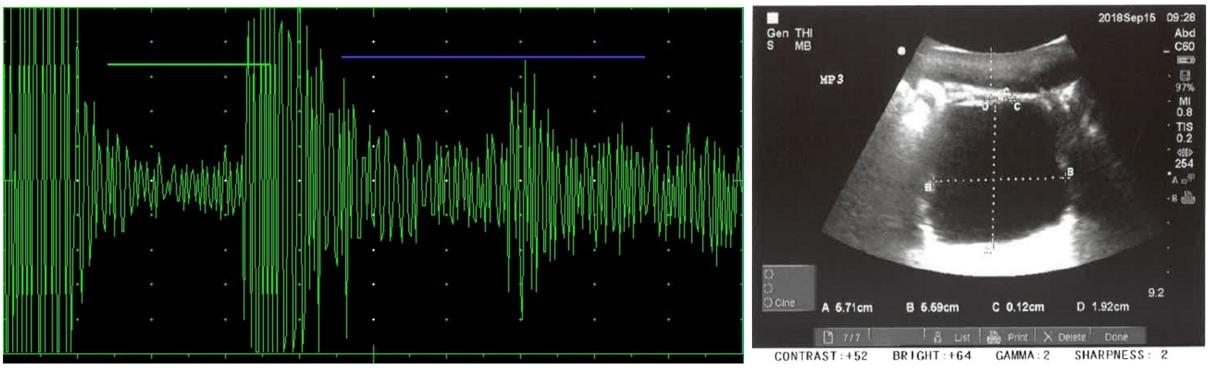

Fig. 14: Volunteer MP3.

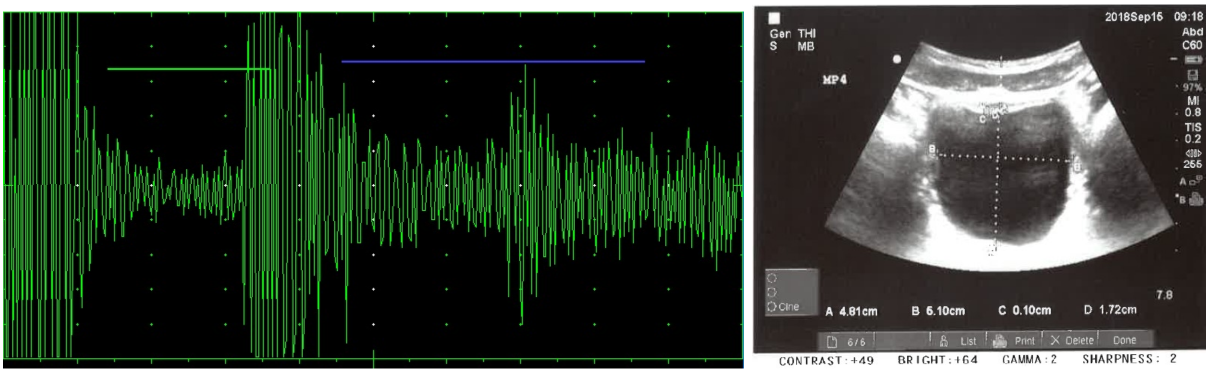

Fig. 15: Volunteer MP3.

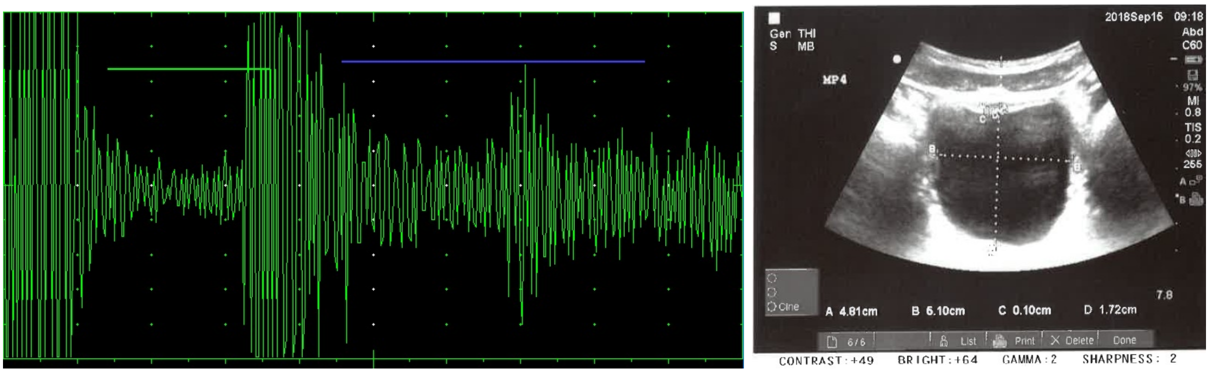

Fig. 16: MP4 second trial.

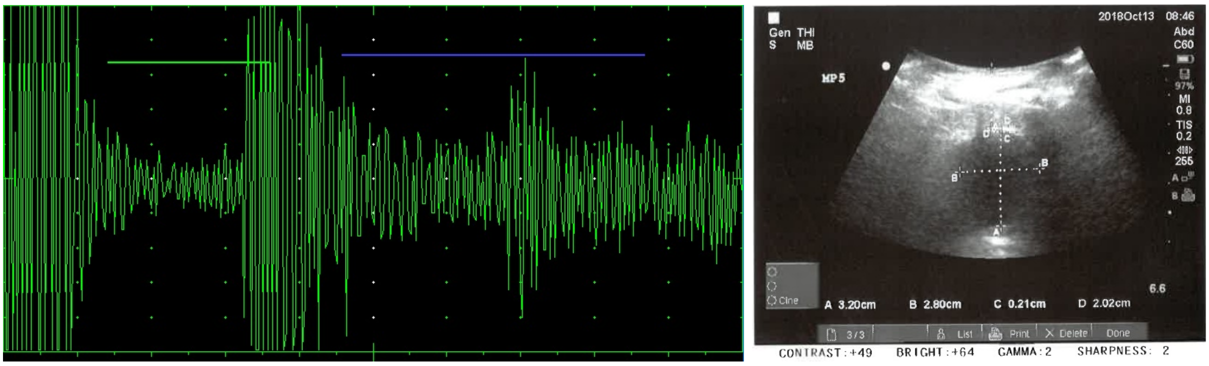

Fig. 17: MP5 first trial.

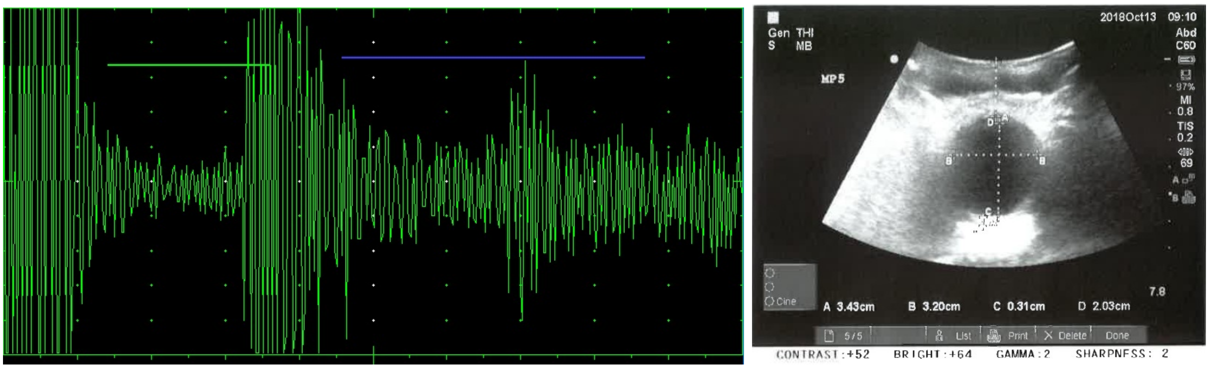

Fig. 18: MP5 second trial.

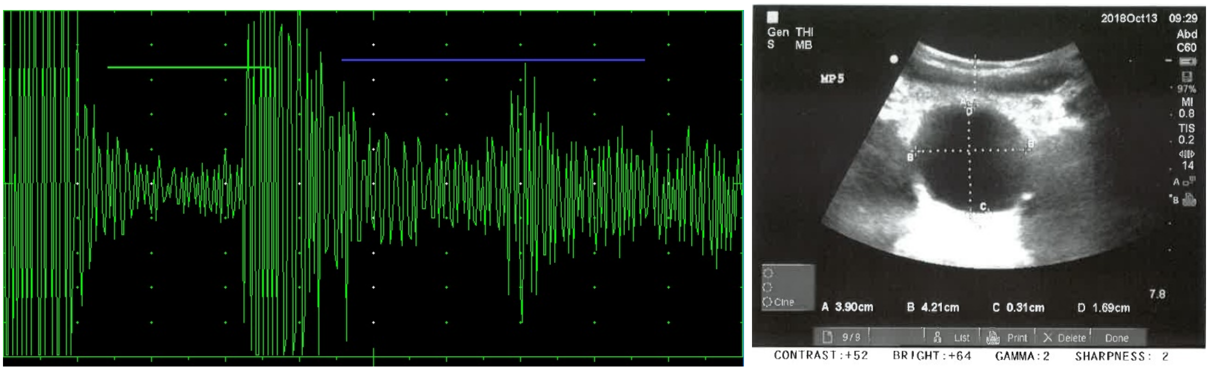

Fig. 19: MP5 third trial.

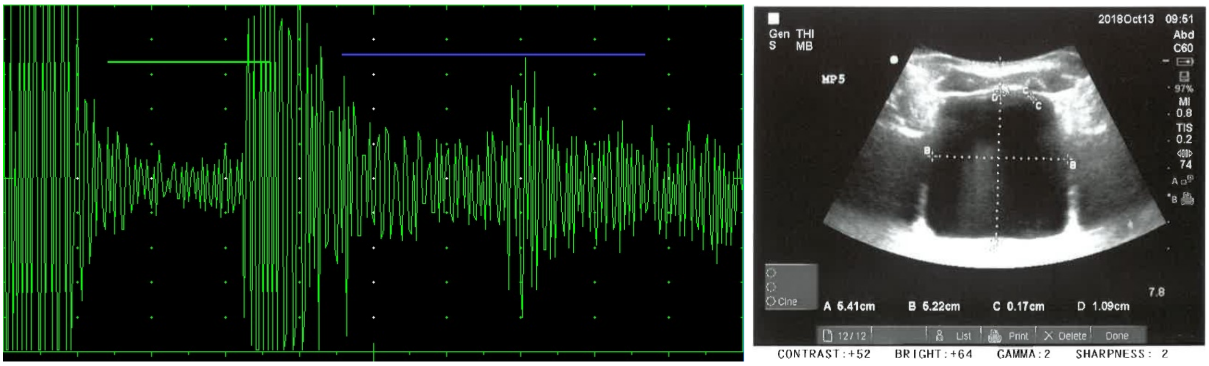

Fig. 20: MP5 fourth trial.

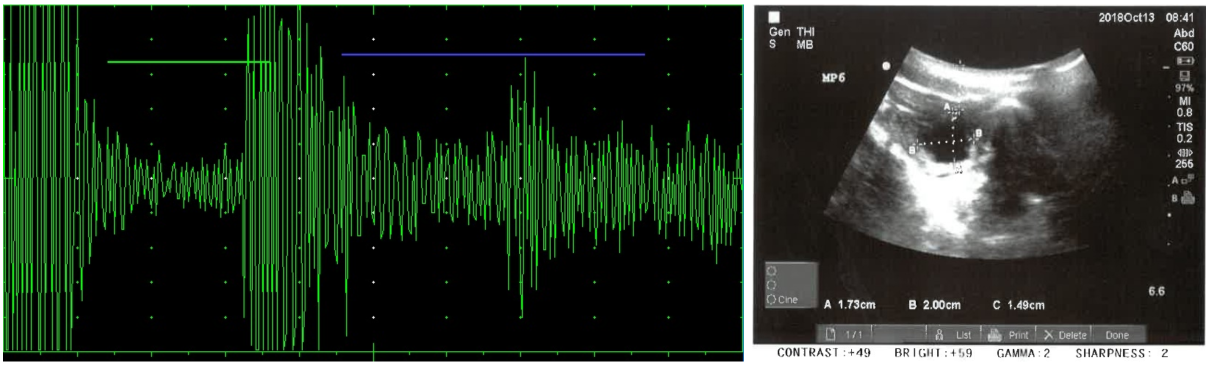

Fig. 21: MP5 first trial.

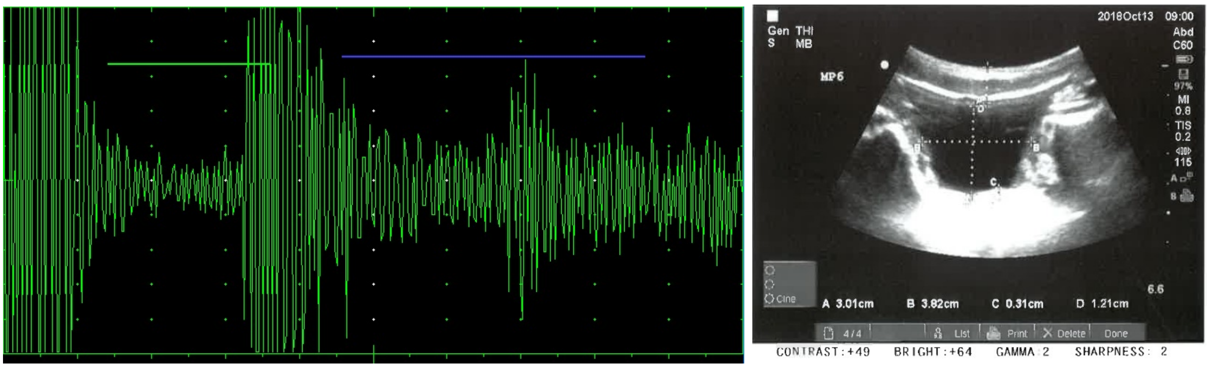

Fig. 22: MP5 second trial.

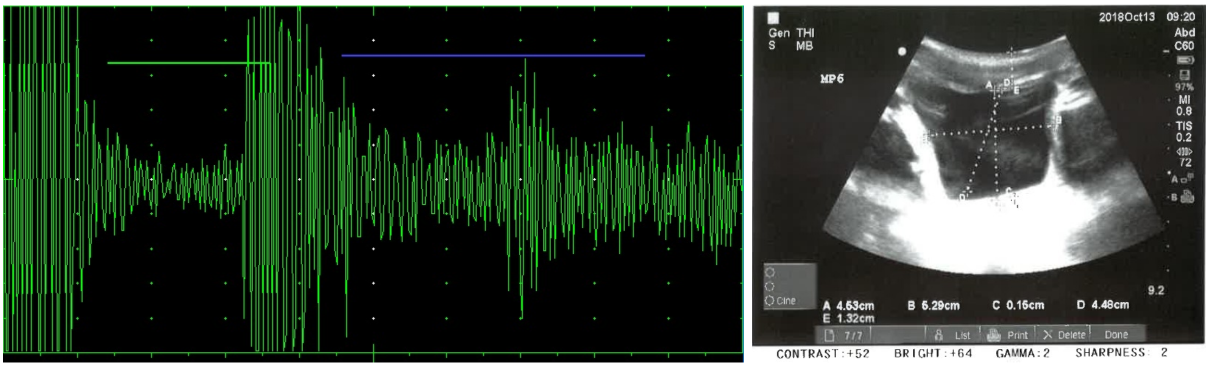

Fig. 23: MP5 third trial.

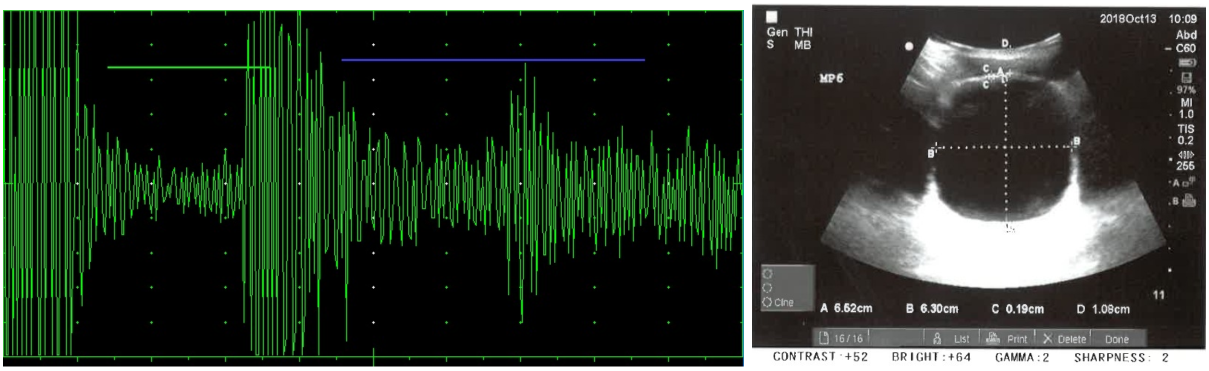

Fig. 24: MP5 fourth trial.

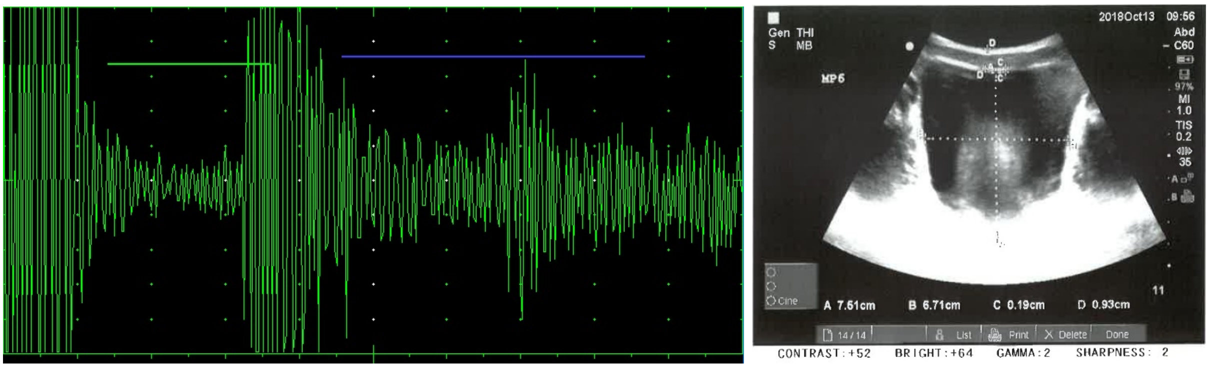

Fig. 25: MP5 fifth trial.

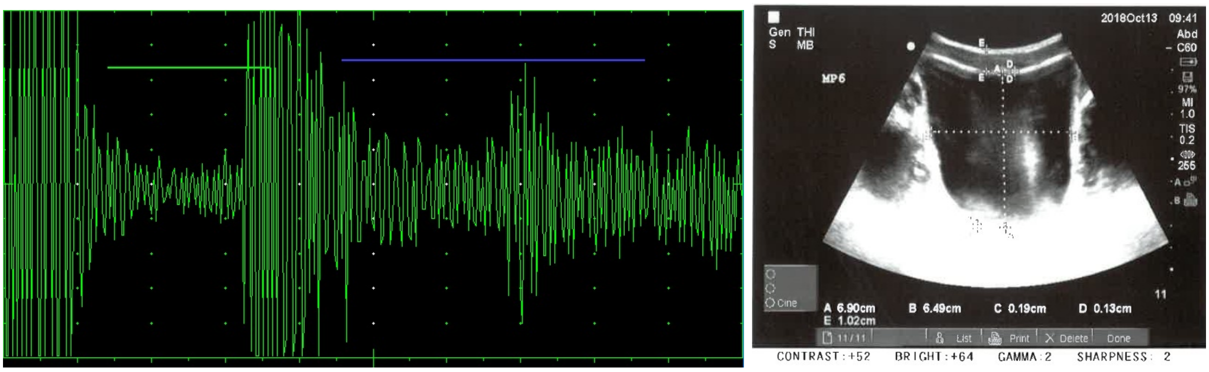

Fig. 26: MP5 sixth trial.

Initial Experience/Evaluation/Comfort

---

1. Initial fitting instructions: Information sheet

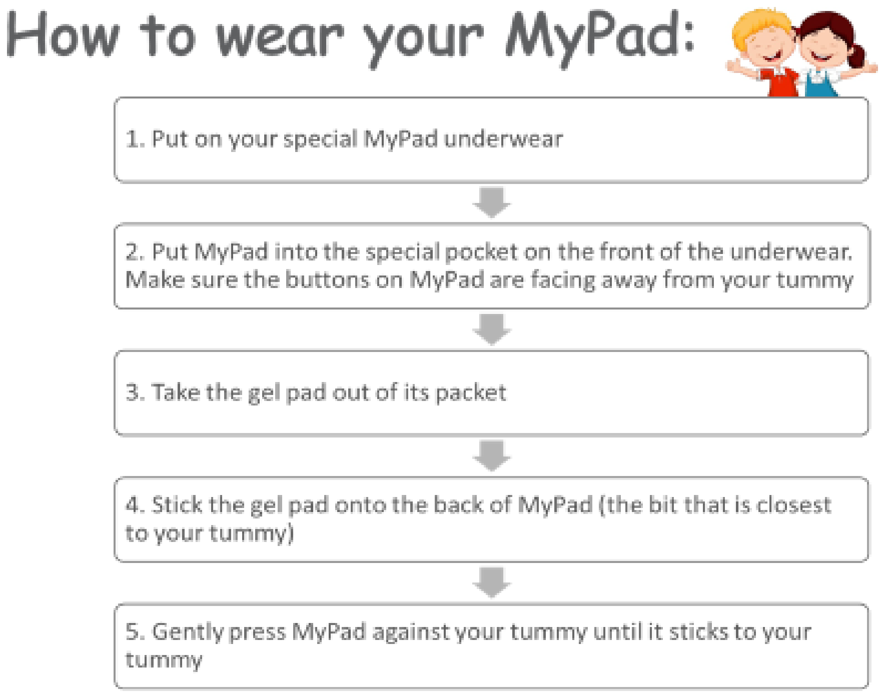

This is where MyPad should stick to your tummy:

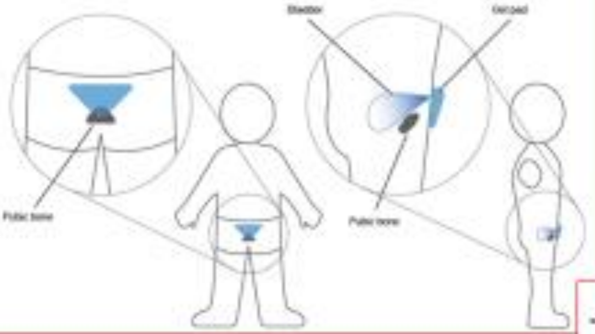

The special underwear will help MyPad to stay in the right place on your tummy

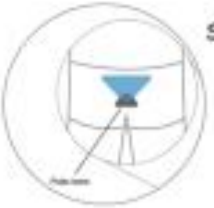

This is how to make MyPad work:

- MyPad (INSERT SYMBOLS)

2. Comfort/fit test protocol

- Walk 15m (any problems?) Yes/No
- Sit with legs open. (any problems?) Yes/No
- Sit with legs crossed (any problems?) Yes/No
- Lie on your right side. (any problems?) Yes/No
- Lie on your left side. (any problems?) Yes/No
- Lay on your front. (any problems?) Yes/No
- Lay on your back. (any problems?) Yes/No

IF you said YES to any of the questions above, please tell us what problems you had:

____
____
____
____
____
____
____
____
____
____
____

What do you like about the MyPAD?

____

____

____

____

____

____

____

What do you NOT like about the MyPAD?

____

____

____

____

____

____

____

____

____

____

____

3. MyPAD Rating Scale **(to be completed at the initial comfort/fit trial and following the overnight trial by the child – with the help of the carer).**

The device makes me feel………….	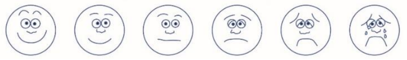
I like the look of it	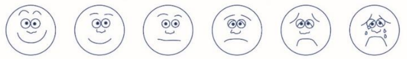
I am conscious/aware of it on my body	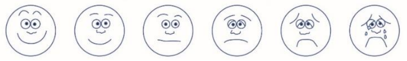
I can relax when I'm wearing it	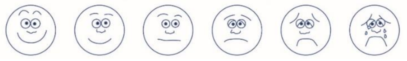
I worry about using it/wearing it	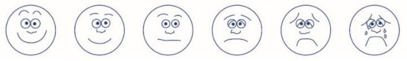
**Is the device:**	
Annoying/irritating to wear?	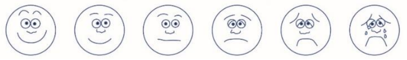
Cool/fashionable to wear?	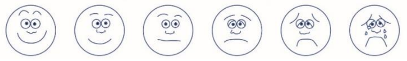
Embarrassing to wear?	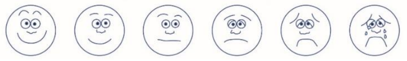
Restricting your movement?	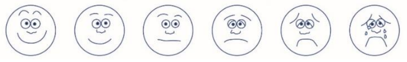

Hidden enough for you?	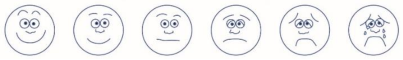
**Does the device:**	
Feel loose?	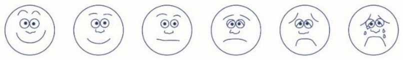
Pull on your clothes?	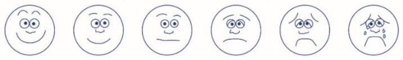
Squash your tummy?	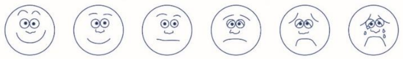
Feel heavy on your tummy?	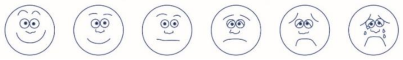
Move differently to your body?	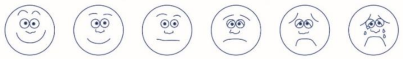
Buzz/tickle?	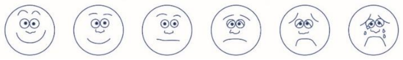
Leave bruises/marks?	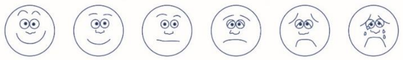
Make your tummy feel damp/clammy?	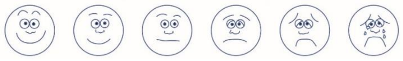
Dig into your tummy?	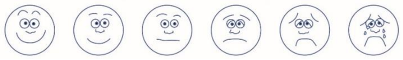
Cause you pain?	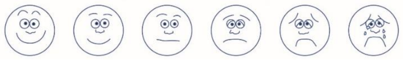

Feel tight?	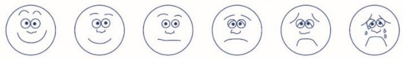
Make your tummy tingle?	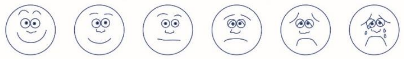
Affect your balance?	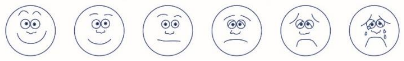
Feel as though it might fall off?	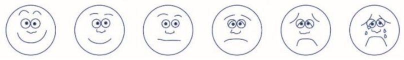
Has the device made moving about difficult?	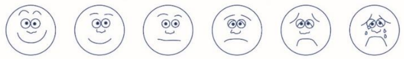
Is wearing the device difficult?	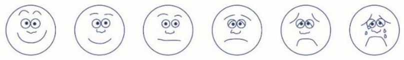
Does your brain have to work hard when you wear the device?	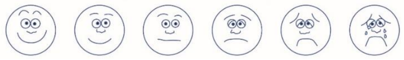
Does the device stay on your tummy where it is supposed to?	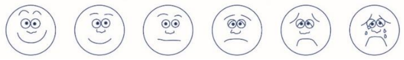
**Does wearing the device:**	
Stop you from moving properly?	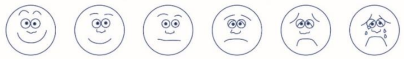
Stop you from doing activities?	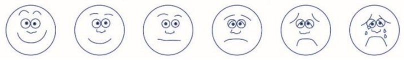
Affect your posture?	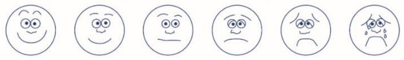

2 Questionnaires for the comfort trials

Get in the way?	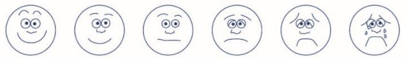
Make your muscles tired?	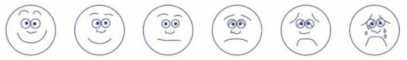
Make you bump into things?	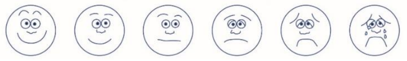
Make you move more or less?	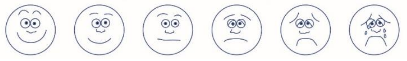
**Do you worry:**	
About damaging the device?	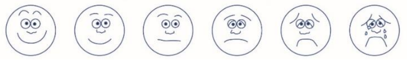
That the device might be dangerous?	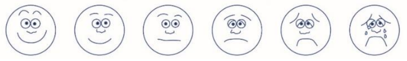
About the device working properly?	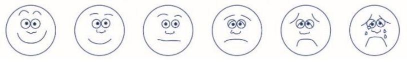
